# Mapping the electrostatic force field of single molecules from high-resolution scanning probe images

**DOI:** 10.1038/ncomms11560

**Published:** 2016-05-27

**Authors:** Prokop Hapala, Martin Švec, Oleksandr Stetsovych, Nadine J. van der Heijden, Martin Ondráček, Joost van der Lit, Pingo Mutombo, Ingmar Swart, Pavel Jelínek

**Affiliations:** 1 Department of Thin Films and Nanostructures, Institute of Physics, Academy of Sciences of the Czech Republic, v.v.i., Cukrovarnická 10, 162 00 Prague, Czech Republic; 2 Department of Chemistry, Condensed Matter and Interfaces, Debye Institute for Nanomaterials Science, Utrecht University, PO Box 80 000, 3508 TA Utrecht, The Netherlands

## Abstract

How electronic charge is distributed over a molecule determines to a large extent its chemical properties. Here, we demonstrate how the electrostatic force field, originating from the inhomogeneous charge distribution in a molecule, can be measured with submolecular resolution. We exploit the fact that distortions typically observed in high-resolution atomic force microscopy images are for a significant part caused by the electrostatic force acting between charges of the tip and the molecule of interest. By finding a geometrical transformation between two high-resolution AFM images acquired with two different tips, the electrostatic force field or potential over individual molecules and self-assemblies thereof can be reconstructed with submolecular resolution.

## Abstract (web summary)

The chemical properties of molecules are largely determined by the distribution of charge across them. Here, the authors demonstrate how the electrostatic force field, originating from the inhomogeneous charge distribution in a molecule, can be measured with sub-molecular resolution.

## Graphical abstract

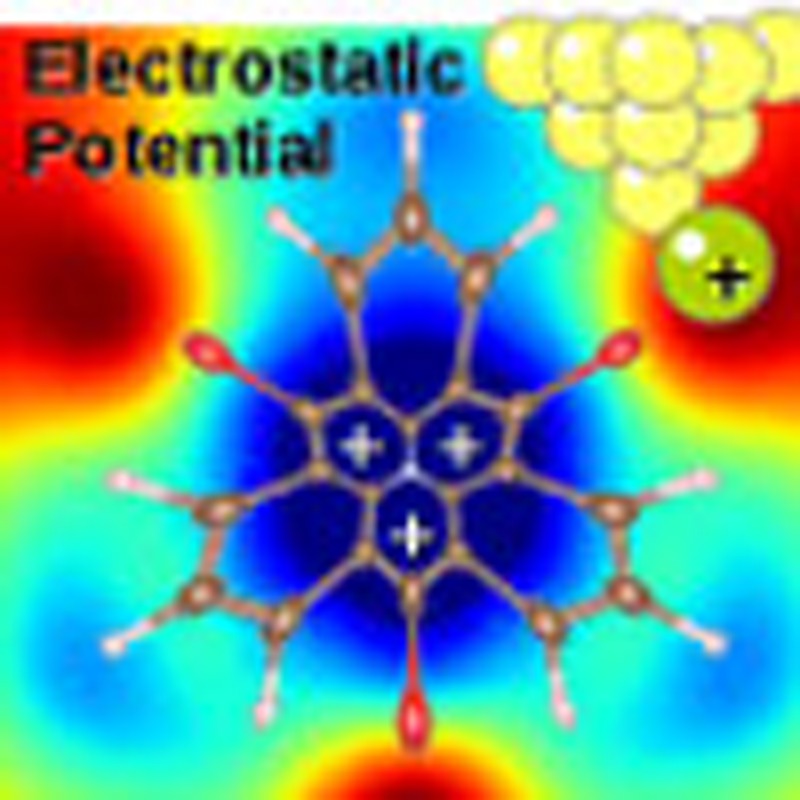

Scanning probe techniques routinely provide detailed information on the electronic and geometric structure of molecules. For example, the frontier molecular orbitals^1^, the chemical structure of molecules^2^,^3^,^4^ and bond orders^5^ can be imaged. The possibility to image molecules^6^ and atomic clusters^7^ with nearly atomic resolution, also at elevated temperatures^8^,^9^,^10^,^11^ provided a great stimulus for surface science^12^,^13^,^14^,^15^,^16^,^17^.

From the perspective of chemistry, the capability to measure the charge distribution of a molecule is extremely useful as this property determines the chemical reactivity of a molecule, as well as many other molecular properties^18^. However, imaging the charge distribution within a single molecule remains a challenge. Thus far, kelvin probe force microscopy (KPFM)^19^ is the only technique able to measure a quantity that is related to the charge distribution of an individual molecule^20^: the local contact potential difference^21^. The acquisition and unambiguous interpretation of KPFM data on the atomic^22^,^23^ and submolecular level is a non-trivial task^20^,^24^. One of the primary difficulties is that there is no clear definition to which physical quantity (electrostatic potential, field or surface dipole and so on) the detected signal should be compared (see, for example, discussion in ref. 25). Furthermore, at the typical tip-sample distances required to obtain submolecular resolution in atomic force microscopy (AFM) images, the measured KPFM signal is governed by the complex interplay of local electrostatic fields of tip and sample, their mutual polarization^26^, mechanical distortions and the conductance due to overlap of molecular orbitals^27^. In this regime, the usual interpretation of KPFM data is longer valid^28^.

Very recently, two alternative techniques, the Scanning Quantum Dot Microscopy^29^ and the kelvin probe force spectroscopy^28^, were introduced. Both methods partially solve the deficiencies of the KPFM method discussed above. Namely, Scanning Quantum Dot Microscopy is able to provide a quantitative analysis of the electrostatic potential, but only in the far distance regime, limiting the spatial resolution. The kelvin probe force spectroscopy method provides high spatial resolution but suffers from the same drawback of ambiguous definition of the observable as KPFM (ref. 28).

As the charge distribution is to be imaged with high-resolution resolution, the use of chemically passivated tips is essential^30^,^31^,^32^. Several different types of forces and processes have been identified to be important for the contrast in AFM images acquired with such tips. These include the Pauli, van der Waals and electrostatic forces, as well as the flexibility of the functionalized tip^2^,^5^,^33^,^34^,^35^,^36^,^37^,^38^,^39^. The latter is especially important to understand the distortions in the appearance of molecules in submolecular resolution images^34^,^36^,^40^,^41^,^42^,^43^,^44^,^45^.

Here we will show that the electrostatic forces acting between probe and an inspected molecule can significantly affect the submolecular contrast. Furthermore, we will show that distortions of the high-resolution images induced by the electrostatic force can be used to map the electrostatic potential of the molecule with submolecular resolution.

## Results

### General considerations

**Figure 1:**
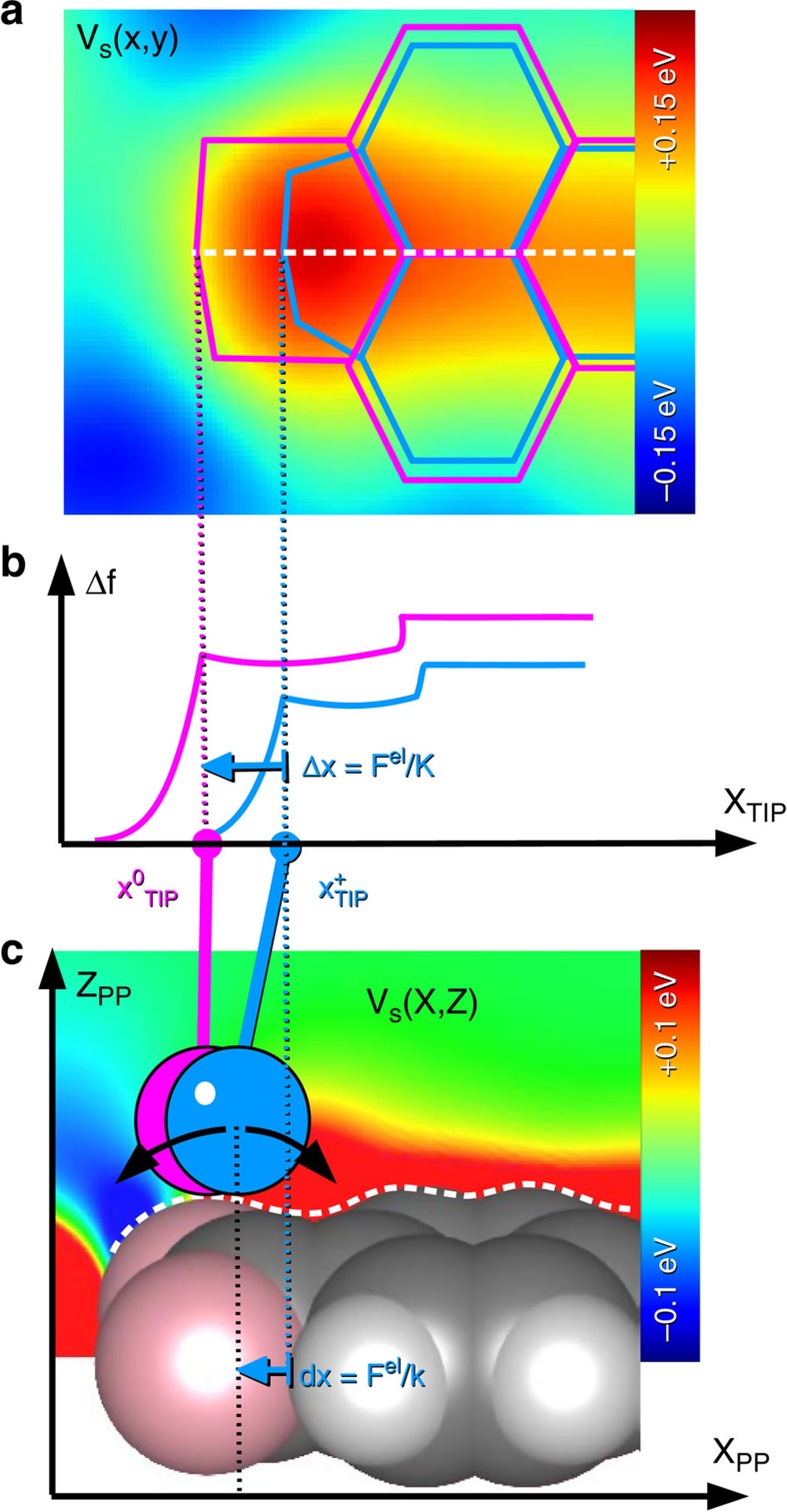
Schematic view of the impact of the electrostatic forces on the high-resolution AFM images. (**a**) Blue and pink lines represent the positions of sharp edges observed in high-resolution AFM images acquired using positively charged and neutral tips, respectively. Corresponding *x*,*y* cut-plane of the Hartree potential *V*_S_(*x*, *y*) above the molecule is displayed in the background. (**b**) Variation of the frequency shift Δ*f* as function of the tip positions *x*_TIP_ for the neutral 
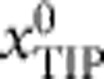
 and positively charged 
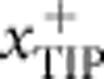
 probe particle. (**c**) The lateral relaxation Δ*x* of the probe particle with (blue) and without (pink) an effective charge on the tip is different above a molecule due to the presence of the electrostatic force. The electrostatic force originates from the interaction between the electrostatic potential of the molecule (*V*_S_) on the surface and the effective charge on the probe particle at its given position *x*_PP_.

To illustrate the central idea behind the method proposed here, we consider imaging a neutral molecule with an inhomogeneous charge distribution with a tip terminated by a positively charged flexible probe particle. The probe particle is attracted to regions of excess electron density, whereas it is repelled from regions that have a positive charge. Consequently, positively/negatively charged areas will appear smaller/larger than they really are with such a tip, as illustrated in Fig. 1. The opposite tendency is true for negatively charged tips. Hence, the distortions in submolecular resolution AFM images acquired with charged tips carry information on the charge distribution within the molecule. Here, we demonstrate how these image distortions can be used to determine the spatial distribution of the electrostatic field above molecules with submolecular resolution. The technique is applied to reconstruct the local electrostatic field of both individual molecules and self-assembled monolayers.

**Figure 2:**
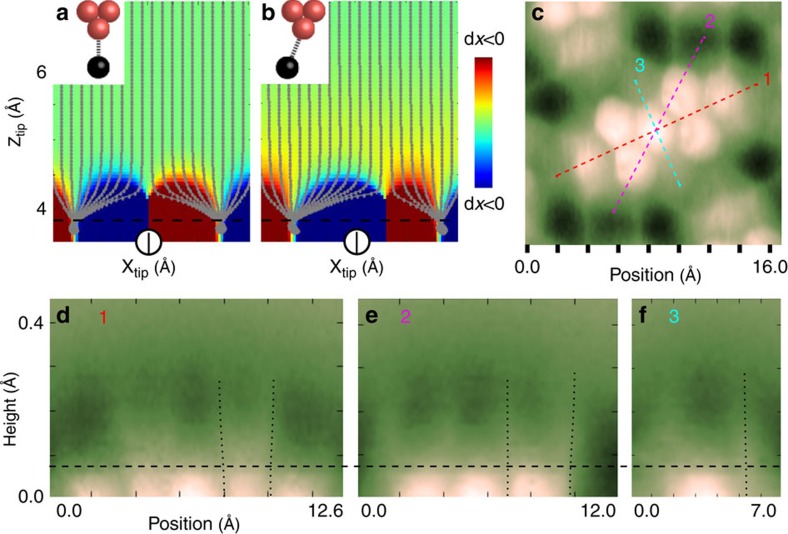
Height dependence of the position of the sharp edges in AFM images. (**a**) Simulated deflections of the probe particle as a function of tip position. Grey lines represent the additional deflection of the probe particle with respect to its optimal configuration in far distance. The deflection of the probe particle to the left and right is indicated by blue and red, respectively. (**b**) Same as **a** but now in the presence of an additional constant lateral force (see Supplementary Fig. 2 and Supplementary Note 2 for more details). (**c**) Constant-height AFM image of PTCDA on Ag(111). Dashed lines with numbers 1,2,3 denote positions of different vertical cut planes. (**d**–**f**) Evolution of Δ*f* with height along the lines shown in **c**. The black dotted lines guide the eye to see relevant edges. The horizontal dashed line indicates the approximate *z*-distance of contrast inversion.

First, let us briefly discuss the origin and character of the apparent bonds or sharp edges in high-resolution AFM/STM images. At close tip-sample distances, the repulsive Pauli interaction induces a significant lateral deflection of the probe particle. There is a discontinuity in the deflection above saddle points of the energy landscape (Fig. 2a,b). The saddle points (sharp edges) are typically present over atoms or bonds at a tip-sample distance where the Pauli repulsion fully compensates the attractive forces. Consequently, the trajectory of the probe particle is split into branches, giving rise to sharp edges^35^,^38^,^43^. Hence, the apparent bonds correspond to saddle points of the potential energy surface experienced by the probe particle at a certain tip-sample distance.

Figure 2a shows the simulated deflections of the probe particle on tip approach over a one-dimensional chain of atoms separated by 2.9 Å (corresponding to the width of a typical benzene ring). The lateral deflection of the probe particle to the left and right is depicted in blue and red, respectively. Note that the trajectories of the probe particle are split into two branches. The deflection depends non-linearly on the tip height. However, the position of the sharp discontinuous boundary between bending left and right, that is, between blue and red regions, does not depend on the tip-sample distance. This is in agreement with our experimental observation that at close tip-sample distances the apparent position of various sharp edges in AFM images of a perylene-3,4,9,10-tetracarboxylic dianhydride (PTCDA) molecules on Ag(111) does not change with distance (Fig. 2c–f). This finding can be rationalized by the fact that the position of sharp edges is determined by the distance where the bifurcation of the probe particle trajectory on tip approach happens. Consequently, while the lateral distortions of the probe particle may be large beyond this point, the lateral apparent position of the sharp edge remains constant.

The total lateral force **F**_tot_ is the sum of van der Waals (**F**_vdW_), Pauli (**F**_Pauli_) and optionally electrostatic (**F**_el_) forces. The presence of the lateral force (**F**_tot_) induces a lateral deflection (d**x**) of the flexible probe particle with respect to the tip position (**x**_tip_), see Fig. 2b. As long as d**x** is small, it is linearly proportional to **F**_tot_ acting on the probe particle, according to Hooke's law: d**x**=**F**_tot_/*K* (refs 34, 39), where *K* is the lateral bending stiffness of the bond between the probe particle and the tip. Variation of the lateral electrostatic force **F**_el_ causes a shift of the characteristic feature at a different lateral tip position (indicated by 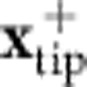) with respect to the position with the absence of the electrostatic lateral force 
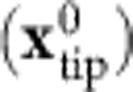
 as shown in Fig. 1. Consequently, the positions of the apparent bonds (**x**_tip_) in high-resolution AFM images do not correspond to their actual positions on the surface.

From the above, it is clear that the apparent shift of the characteristic features (Δ**x**) in AFM/STM images, therefore, carries information about the lateral forces **F**_tot_ with atomic resolution. The apparent shift is linearly related to the deflection of the probe particle from the tip base: Δ**x**=*γ*d**x**=*γ*(**x**_tip_−**x**_PP_), see Fig. 1. Here, **x**_PP_ denotes the actual position of the probe particle and *γ*≈2. A detailed quantitative analysis including the definition of the γ coefficient can be found in Supplementary Note 1. In the following discussion, we will express everything in an experimentally observable Δ**x** instead of in d**x**. In this notation, Δ**x** is linearly proportional to the lateral force: Δ**x**=**F**_tot_/*k*, where *k*=*K*/*γ* is an effective lateral stiffness.

In our analysis of the electrostatic field, we will use the differences in the apparent positions of sharp contours recorded with two different tips. Specifically, we extract and compare the apparent positions of the same contour feature (for example, a particular atom vertex or bond edge) from two high-resolution AFM images obtained with different tips or scanning conditions (labelled tip *A* and tip *B*) at approximately the same tip-sample distances. The apparent position of features acquired with the different tips are indicated by **x**_tip,A_ and **x**_tip,B_, respectively. In the following, we are interested in the relative difference of the apparent positions *δ***x**=**x**_tip,A_−**x**_tip,B_. Because we measure the same object on the surface, the real position of any atom or bond that corresponds to a particular contour feature is the same for both images. Therefore, *δ***x** can be expressed as the difference between the image distortions in the two images of the same object:


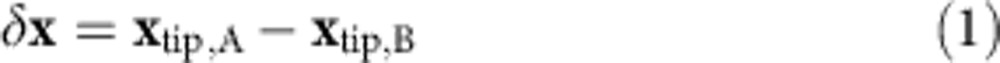



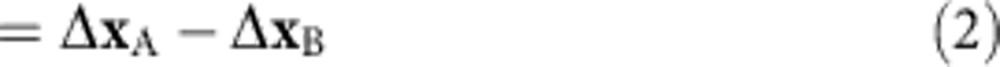



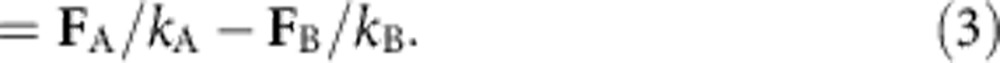


Here, **F**_A_ and **F**_B_ are the total lateral force acting on the probe particle in case *A* and *B*, respectively. As shown in the Supplementary Note 1, only the van der Waals and electrostatic force components of the total force contribute to the apparent lateral distortions Δ**x**. The reason why **F**_Pauli_ can be ignored is connected with the rather abrupt onset of the Pauli repulsion, as we explain in Supplementary Fig. 1 using our hard-sphere model. The distortion then depends linearly on the lateral van der Waals and electrostatic forces (Supplementary Note 2 and Supplementary Fig. 2). In the following, we therefore use the following expression for the differences in the lateral distortions:


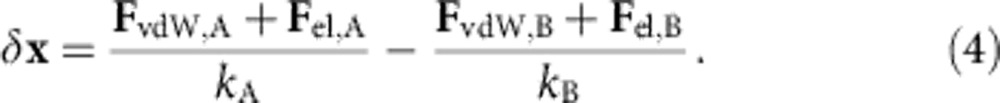


In this general form, there is unfortunately no clear way how to attribute partial relative distortions to each force component, for example, to determine the lateral electrostatic force field **F**_el_ component only from the high-resolution images. However, under certain assumptions and/or with the help of numerical simulations this problem can be circumvented.

We will now discuss an approach to extract the lateral electrostatic force field component from equation (4). A detailed discussion is given in Supplementary Note 3. In general, the effective stiffness (*k*) and charge (*Q*) of the tips, as well as the van der Waals contributions are different for each tip. The charge of the probe particle depends on the configuration and chemical nature of the metal apex^46^, as well as how the Xe/CO is coordinated. Hence, even tips terminated with the same species can have a different charge. First, we will consider the simplest case: two high-resolution images are acquired with (nearly) identical tips, for example, Xe-terminated metallic tips, differing only in their effective charge. In principle, they can have different dipoles instead of effective charges, but this would not change the conclusion drawn later (Supplementary Note 4 and Supplementary Fig. 3). In this case, the following approximations hold: (i) the effective lateral stiffness *k* of both tips is identical or very similar (that is, *k*_A_≈*k*_B_=*k*); (ii) the lateral components of the van der Waals forces for tip *A* and *B* are also almost identical at a given tip-sample distance. Since both tips are used to image the same object, the surface electrostatic field (**E**_S_) must be the same in both images. Under these approximations, equation (4) simplifies to:


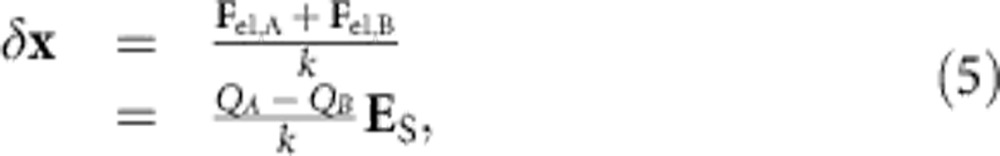


where *Q*_*A*_, *Q*_*B*_ are the effective charges of tips *A* and *B*, respectively. This equation shows that we can obtain quantitative information about **E**_S_ directly from the difference in the image distortions. The only parameters that are needed are the effective lateral stiffness *k* and the difference between the effective charges *Q*_*A*_ and *Q*_*B*_. These can be estimated for each tip from a direct comparison between experimental and simulated high-resolution AFM/STM images^35^,^47^ (for more details see Supplementary Method: correlating the experimental and theoretical data sets to obtain the probe characteristics *K* and *Q* and Supplementary Fig. 4). Alternatively, *k* can be obtained directly from experimental measurements^48^.

Next, we consider a more general case where the van der Waals contribution for the two tips is significantly different but one of the tips is neutral, that is, *Q*_B_≈0. This would correspond to the situation where macroscopically different tips, possibly with different tip termination, are used. In this case, we obtain the following relation for the surface electrostatic field **E**_S_ (for details see Supplementary Note 3):


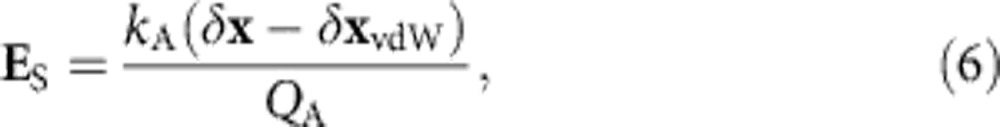


where *δ***x**_vdW_=**F**_vdW,A_/*k*_A_−**F**_vdW,B_/*k*_B_. The differences of the van der Waals deformation field components (*δ***x**_vdW_) can be estimated from numerical simulations, as discussed later. We note here that the effect of the van der Waals contribution is indispensable only at the periphery of the molecule. Although it may be difficult to extract absolute values in this scenario, the overall shape of the electrostatic field is preserved.

To test our approach to measure the electrostatic field with submolecular resolution, we performed two different sets of experiments. First, we studied a densely packed self-assembled layer of PTCDA on Ag(111) with two differently charged but otherwise similar Xe-terminated tips. Second, we studied individual 1,5,9-trioxo-13-azatriangulene (TOAT) molecules on Cu(111) with a neutral CO terminated tip and a positively charged Xe tip.

### Molecular layers of PTCDA on Ag(111)

In the case of densely packed self-assembled molecular layers, the van der Waals force component varies slowly. In addition, the effective stiffness *k* for different tips typically has similar values (see later). Therefore, the term *δ***x**_vdW_ in equation (6) can be neglected and the surface electrostatic field is given by *δ***E**_S_=*k*_A_*δ***x**/*Q*_A_.

**Figure 3:**
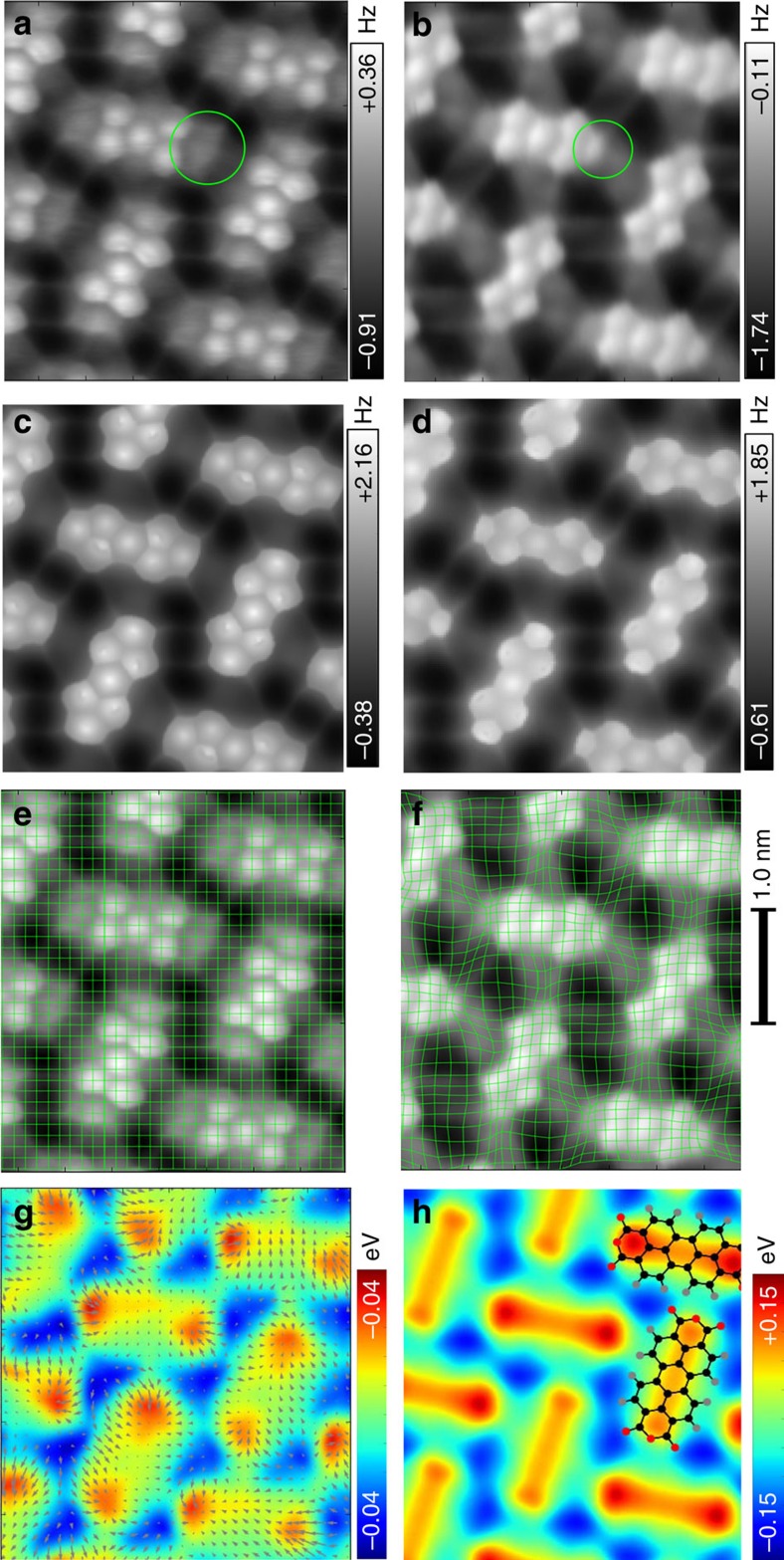
Determining the electrostatic field above a close-packed PTCDA layer. (**a**,**b**) experimental high-resolution AFM images of a self-assembled monolayer of PTCDA deposited on Ag(111) obtained with two different Xe tips. (**c**) simulated AFM image using an effective charge *Q*=0.0e and the effective lateral stiffness *k*=0.16 Nm^−1^. (**d**) same as **c** but with *Q*=+0.3e and *k*=0.20 Nm^−1^. (**e**,**f**) the experimental images superimposed with a deformation grid defined by comparing the corresponding sharp features between the two images in **a** and **b**. (**g**) electrostatic potential calculated from the deformation field (grey arrows). (**h**) calculated Hartree potential from DFT simulations 3.0 Å above the molecular layer.

Figure 3a,b show two constant-height AFM images of a self-assembled monolayer of PTCDA on Ag(111) acquired with a neutral and positively charged Xe tips at the same tip-sample distance (for more details see Methods section). Note that the apparent size of the anhydride groups, indicated by the green circles, is different in the two images. We attribute these differences to a repulsive electrostatic interaction between the positively charged Xe tip and the positively charged anhydride groups^49^. This assignment is supported by a very good agreement between experimental and simulated AFM images of PTCDA/Ag(111) with different effective charges on the Xe tip (Fig. 3c,d). The determined values for *k*_A_, *k*_B_, *Q*_A_ and *Q*_B_ are: 0.16 Nm^−1^, 0.20 Nm^−1^, 0.0 e and +0.3e, respectively, as shown in Supplementary Fig. 4.

The abundant presence of sharp features in the AFM images allows us to use an automatic computer algorithm to determine the differences in the image distortions, that is, *δ***x**. First, two AFM images are brought into register. Subsequently, the distortion field is found by comparing the corresponding sharp features between the two images. The algorithm is based on matching small regions of the two images. The image is divided into small tiles, in our case regularly distributed circular areas with a diameter of the characteristic image feature (for example, C–C bonds, carbon hexagons and so on). These circular areas are each matched to the other image by moving them laterally, searching for maximum correlation. The resulting shift vectors represent a good approximation of the distortion between the two images, and serve as input for the electrostatic potential determination. The green grids plotted in Fig. 3e,f visualize the determined deformation.

As argued above, the as-obtained deformation field (grey arrows in Fig. 3g) is linearly proportional to the lateral electrostatic field above the molecular layer, with proportionality constant *k*_A_/*Q*_A_. The electrostatic potential obtained from the experimental images is shown in Fig. 3g and is in very good agreement with the electrostatic potential as calculated from density functional theory (DFT; Fig. 3h). The absolute magnitude +0.04 to −0.04 eV of the electrostatic field as determined experimentally is approximately three times smaller than estimated from DFT calculations. This discrepancy can be attributed to several effects, such as uncertainties in the absolute tip-sample distance where the electrostatic potential is measured; in the values of the effective charge *Q* and lateral stiffness *k*; in the finite oscillation amplitude and so on. We will address the limits of the method and their possible solutions later (see Discussion).

From the correlation analysis of the experimental and theoretical AFM images of PTCDA (Supplementary Fig. 5), we can estimate the uncertainties in *Q* and *k*. For the neutral tip, the maximum correlation is well defined within ±0.05e and ±0.08 Nm^−1^. However, for the positively charged tip there are multiple *Q*-*k* combinations that provide a similar correlation between experiment and theory. As the scaling term of the vector field is the ratio *Q*/*k*, we can estimate the systematic error from its variation. By selecting the different favourable *Q*,*k* pairs, we find a systematic error of approximately 20%. It is important to note that this uncertainty only affects the absolute values, that is, the relative variation of the electrostatic potential is correct.

### Single TOAT molecule on Cu(111)

**Figure 4:**
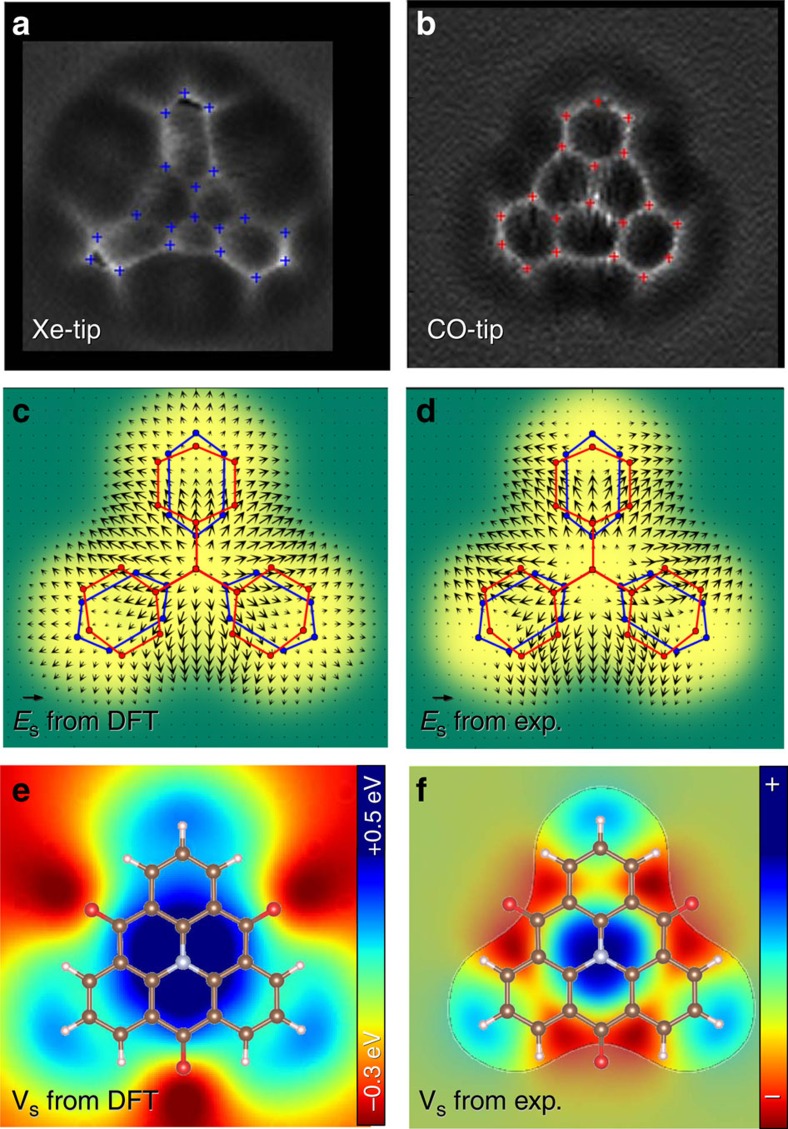
Determining the electrostatic field above an individual molecule. (**a**) High-pass filtered constant-height AFM images of a TOAT molecule on Cu(111) acquired with a Xe tip. Crosses indicate characteristic vertices. (**b**) Same as **a** but measured with a CO tip. (**c**) electrostatic force field calculated from DFT. (**d**) experimentally determined electrostatic force field obtained after subtraction of the van der Waals component from the deformation field obtained from the images shown in **a** and **b**. (**e**) calculated Hartree potential; (**f**) electrostatic potential calculated from the experimental deformation field shown in **d**.

As the second example, we studied individual TOAT molecules, since they have a highly non-homogeneous charge distribution. The central N atom donates an electron to the delocalized π-system and is thus positively charged. In contrast, the three ketone groups at the edge of the molecule withdraw electron density from the ring system and therefore have a partial negative charge. High-pass filtered constant-height AFM images of a TOAT molecule on Cu(111) acquired with CO and Xe tips are shown in Fig. 4a,b, respectively. There are significant differences between images acquired with the two different tip terminations. The central region of the molecule appears smaller while the peripheral benzene rings are elongated for images acquired with a Xe-terminated tip compared to images obtained with a CO tip. Again, this effect is attributed to a repulsive interaction between the positively charged Xe tip and the positively charged central area of the molecule. This effect is reproduced by our simulations for a Xe tip with an effective charge *Q*=+0.3 e and lateral stiffness *k*=0.24 Nm^−1^. Similarly, we found the best match between experimental and theoretical AFM images acquired with the CO tip with an effective charge *Q*=0.0e and lateral stiffness *k*=0.24 Nm^−1^ (details can be found in Supplementary Fig. 5). For individual molecules, positions of vertices were determined manually (blue and red markers in Fig. 4a,b). The deformation field can be obtained by alignment of corresponding vertices (*δ***x**) in the two AFM images using interpolation by radial basis functions and exploiting the threefold rotational symmetry of the TOAT molecule (see Supplementary Method: estimation of the image distortion from high-resolution AFM images of TOAT molecule and Supplementary Fig. 6). The obtained deformation field shown in Supplementary Fig. 7 is directly proportional to total lateral force field.

Here, the estimated lateral force field also contains the van der Waals force component, which can be determined with the help of numerical modelling^34^. Thus the electrostatic field **E**_S_ can be reconstructed from equation (6) using the fitted lateral stiffness (*k*_A_, *k*_B_) and differential van der Waals deformation field *δ***x**_vdW_ (details of how δx_vdW_ was subtracted are provided in the Supplementary Method: subtraction of van der Waals component from distortion field on TOAT). Figure 4d shows the as determined electrostatic field while its calculated counterpart is given in Fig. 4c. The agreement between theory and experiment above the molecule is again very good. Note that the method cannot provide resolution outside of the molecule (green area in Fig. 4c,d), due to the lack of sharp features in this region. Hence we nullified the obtained electrostatic field in this area.

For the TOAT molecules, we obtained the experimental electrostatic potential *V*_S_ by determining the derivative of the electrostatic field **E**_S_. The resulting electrostatic potential, shown in Fig. 4f, matches the calculated electrostatic field over the molecule including a complex charge distribution on the benzene lobes. The charge distribution near the oxygen atoms can not be described properly due to the lack of sharp features in this area. In the TOAT case, our method cannot reliably quantify the absolute magnitude of the electrostatic potential due to uncertainties associated with subtraction of the vdW force field and the absence of sharp features outside the molecule. We decided not to provide a quantitative comparison of the electrostatic potential *V*_S_ to avoid an over-interpretation of our method.

## Discussion

We will now discuss several important aspects of the method to facilitate its assessment. The resolution of our method is directly connected to the requirement of having sharp edges in images originating from a saddle point of the potential energy surface. Therefore, the method can only be used to determine the electrostatic field at close tip-sample distances where such features are present.

As discussed above, the position of the sharp edges remains practically constant in the close distance regime. This has two important consequences. First, the position of sharp edges is determined by a bifurcation (cusp) in the probe particle trajectories upon tip approach. The deflection of the probe particle beyond this branching point does not further affect the apparent position of the edges in the images. Consequently, only the value of the lateral spring constant *k* at the tip-sample distance where the branching occurs will influence the results. Therefore, variations of the lateral stiffness *k* with tip-sample distance^39^ do not affect the analysis. Second, our method can only map the electrostatic field at the height where the trajectory of the probe particle branches.

It is important to note that sharp edges are visible in both simultaneously acquired AFM and STM channels, as shown in Supplementary Fig. 8. In addition, sharp features are also present in high-resolution STM (ref. 3) and IETS-STM (ref. 4) images. Hence, in principle, our method can also be applied to such data.

The possibility to extract quantitative information depends critically on several factors. First, uncertainties in the values of *k* and *Q* could potentially be eliminated by acquiring two images with the same functionalized tip, the charge of which can be effectively modified by other means (for example, by some oxidation/reduction process of the moiety attached to the tip). A search for new functionalized tips with the possibility to modulate an effective charge without loosing its mechanical stability is the subject of current investigations. Alternatively, one can try to reduce the uncertainties in *k* and *Q* by, for example, using more sophisticated algorithms for image analysis. The simulation of the electrostatic interaction can be further improved by implementation of a more realistic charge distribution on the probe particle using dipole/quadrupole or even the charge distribution obtained directly from *ab initio* calculations^50^.

In conclusion, we showed that the electrostatic interaction between the probe and a molecule on the surface affects distortions in high-resolution images. In particular, the electrostatic field originating from polar molecules can be mapped with high resolution by analysing the differences in the distortions in images acquired with differently charged tips. The arguments and results presented above demonstrate the background, advantages and limitations of the method to probe the electrostatic potential of molecules with submolecular spatial resolution. The main advantages of this method are the clear relation between the physical observables and the electrostatic field, the high spatial resolution and its applicability to STM and AFM images. In addition, it offers the prospect of extracting quantitative information. Here, we applied the method for molecules, but it can be easily extended to surfaces and surface defects (for example, impurities, vacancies, subsurface defects and so on). As such, it constitutes a valuable complementary tool to existing techniques.

Finally we would like to stress that the general idea behind the technique can be applied to any lateral force acting on the last atom of the tip (the probe particle). Consequently, new potential applications can be envisaged, such as imaging the electrostatic field of the probe itself or that of excited molecules. In addition, it may be possible to map molecular magnetic field as well.

## Methods

### AFM/STM measurements of PTCDA/Ag(111)

The PTCDA on Ag(111) experiments were carried out with a Specs LT STM/AFM with a commercially available Kolibri sensor, operating at ∼1.2 K in ultra-high vacuum. Kolibri sensor parameters used in experiment are: *f*_0_≈985,387 Hz, *Q*≈230,000 and *A*≈70 pm.

The Ag surface was cleaned by repeated cycles of sputtering (Ar^+^, *p*Ar≈5 × 10^−6^ mbar, 10 min) and annealing (≈800 K, 5 min). PTCDA was evaporated in ultra-high vacuum (*P*<1.5 × 10^−9^ mbar) for 4 min from a crucible thermally heated to ≈673 K. Evaporation was performed ≈10 min after the final annealing of the Ag sample with no post evaporation annealing. Xe (99.99% purity) was deposited on the cold sample (T<10 K) by opening shutters for ≈14 s to *p*_Xe_=5 × 10^−7^ mbar. The tip was functionalized in two steps. First, a metal terminated tip was obtained by few nm dipping of the sensor into the clean Ag surface with a ≈2 V bias pulse. Second, Xe-terminated tip was obtained by spontaneous picking up a Xe atom from a Xe island by the metal terminated tip, while scanning in STM mode (0.1 V, 10 pA).

The acquisition of the three-dimensional (3D) force maps was done automatically, by measuring a sequence of constant-height images and changing the tip-sample separation in between the subsequent images. Apart from the frequency shift, tunnelling current, dissipation and also the amplitude channels have been recorded simultaneously. The step in *z* was chosen to be in the order of picometres and positive, that is, increasing the tip-sample distance.

Images acquired with different tips were aligned vertically using the following procedure. First, for each tip a data cube with simulated 3D frequency shift values is generated for a particular set of *k* and *Q* values. The offsets in *z*-distance of the experimental and theoretical data sets are then determined by aligning the *z*-position of the frequency shift minimum for the centres of the molecules. Once this information is available for each tip, images corresponding to approximately the same tip-sample distance can be selected.

### AFM/STM measurements of TOAT/Cu(111)

Individual TOAT molecules on Cu(111) with a neutral CO terminated tip and a positively charged Xe tip were imaged using a Scienta-Omicron LT STM/AFM with a commercially available Qplus sensor, operating at ∼4.6 K in ultrahigh vacuum with an average pressure of 5 × 10^−10^ mbar. The baked qPlus sensor (3 h at 120 °C) had a quality factor of *Q*=30,000, a resonance frequency of *f*_0_=25,634 Hz and a peak-to-peak oscillation amplitude of approximately 2 Å.

A Cu(111) crystal surface was cleaned with several sputter and anneal cycles before inserting it in the microscope head. The TOAT molecules were thermally evaporated onto the cold surface using an e-beam evaporator (Focus GmbH). For STM imaging, the bias voltage was applied to the sample. After approaching the tip to the surface, an atomically sharp metal tip was prepared by controlled crashes into the copper surface and bias pulses. Each chemically passivated tip was prepared by subsequent pick-up of either a Xe atom or CO molecule^2^,^51^,^52^,^53^. After a free-lying TOAT molecule was located on the surface, the tip was left in tunnelling contact (*I*=10 pA at *V*=0.1 V) and allowed to relax for 12 h to minimize drift and piezo-creep. All AFM images were acquired in constant-height mode. After each AFM image, the STM feedback loop was enabled for 2 s to further minimize tip-sample drift. A complete stack of images resulting in a 3D force grid took ∼13 h to acquire.

### DFT calculation of PTCDA/Ag(111)

We used a pre-optimized herringbone structure of PTCDA molecules on Ag(111) surface^54^ consisting of two molecules in the unit cell and a slab of 3 Ag layers (99 Ag atoms). The Hartree potential used for generating the theoretical electrostatic force field^35^ was obtained from self-consistent total energy DFT using the Vienna ab initio simulation package^55^ with generalized gradient approximation based functional PW91 (ref. 56) and projector augmented-wave method^57^. Plane wave basis set was chosen with *E*_cut_=396 eV.

### DFT calculation of TOAT/Cu(111)

Total energy DFT calculations were performed using the FHI-aims code^58^. We used a 6 × 6 supercell made of four Cu layers to describe the Cu(111) surface. The TOAT molecule was placed on the surface with the N atom in a top position. This position was chosen based on the experimental findings. All the atoms except the two bottom Cu layers were relaxed until the remaining atomic forces and the total energy were below 10^−2^ eV Å^−1^ and 10^−5^ eV, respectively. A Monkhorst-Pack grid of 2 × 2 × 1 was used for the integration in the Brillouin zone. All the calculations were carried out at the GGA-PBE level^59^ including the Tkatchenko-Scheffler treatment^60^ of the van der Waals interaction. The use of van der Waals interactions was necessary to correctly describe the interaction between the molecule and surface. The basis set, pseudo-potentials, integration grids and Hartree potential accuracy were specified using the ‘tight' settings. For species like H, O, N the basis set level was set to ‘tier 2' while for Cu a first tier was used. Note that a ‘tier' represents a single set of radial functions added to the minimal basis to effectively describe the chemical bond.

### AFM simulations

To calculate high-resolution AFM images we used a home built AFM simulation toolkit^34^,^35^ (avaible opensource at https://github.com/ProkopHapala/ProbeParticleModel; see also webpage http://nanosurf.fzu.cz/ppr/). We used default parameters of pairwise LJ potentials^34^. The optimized structures and corresponding surface Hartree potentials were obtained from fully relaxed total energy DFT simulations of the system, see above. The effective tip charge *Q* and lateral stiffness *k* of probe particle are a free input of the model. The positions of the *Δf(z)* minima in the centres of the molecules were taken as the reference points in *Z*, for both the experiment and theory.

### Processing of experimental data and matching to simulation

Iterative algorithm for registration of experimental AFM/STM images using linear correlation is described in the Supplementary Method: data set registration procedure and Supplementary Fig. 9. The estimation of the effective stiffness *K* and effective charge *Q* is described in the Supplementary Method: correlating the experimental and theoretical data sets to obtain the probe characteristics *K* and *Q* and Supplementary Fig. 4 for PTCDA and Supplementary Fig. 5 for TOAT. The evaluation of the electrostatic potential by fitting its derivatives to image distortions is described in the Supplementary Method: evaluation of the electrostatic potential from the distortion vector field.

### Data availability

[Supplementary-material S1]The data that support the findings of this study are available from the corresponding authors on request.

## Additional information

**How to cite this article:** Hapala, P. *et al*. Mapping the electrostatic force field of single molecules from high-resolution scanning probe images. *Nat. Commun.* 7:11560 doi: 10.1038/ncomms11560 (2016).

## Supplementary Material

Supplementary Information Supplementary Figures 1-9, Supplementary Notes 1-4, Supplementary Methods and Supplementary References
