# Supplementary material for: Mapping the electrostatic force field of single molecules from high-resolution scanning probe images
Source: Nat Commun. 2016 May 27;7:11560. doi: 10.1038/ncomms11560 (PMC4894979; doi:10.1038/ncomms11560)
Supplement: Supplementary Information — Supplementary Figures 1-9, Supplementary Notes 1-4, Supplementary Methods and Supplementary References [file ncomms11560-s1.pdf]

SUPPLEMENTARY FIGURES

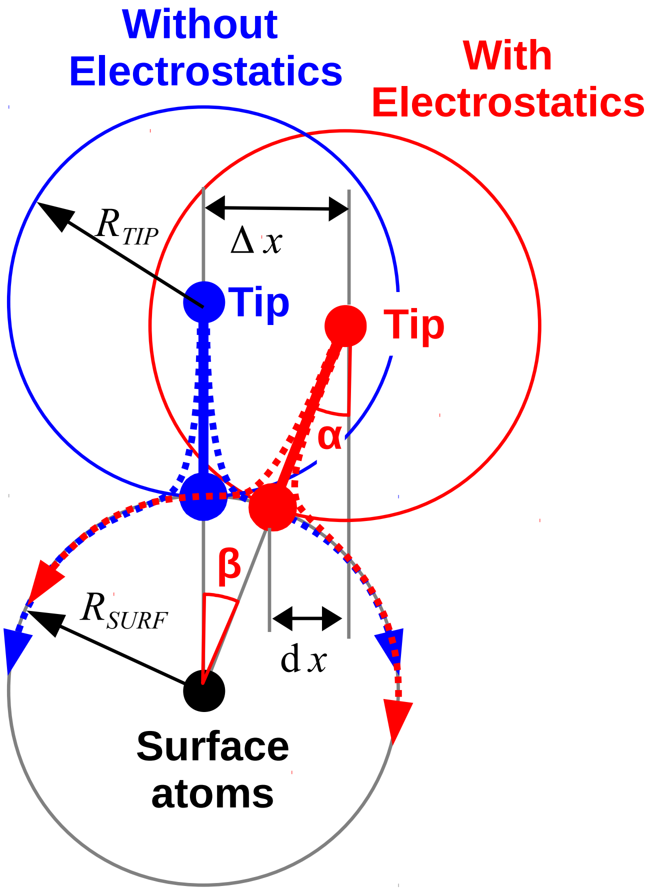

Supplementary Figure 1. **Hard sphere model of probe particle deflection after contact.** Blue is situation without additional lateral force F_x, while red is situation with added lateral force. Circles indicate boundaries of impenetrable sphere which probe particle cannot pass. Arrows suggest trajectories of probe particle upon approach. The picture should illustrate geometrical relations between apparent shift of feature position Δx and deflection of probe particle dx at critical point of relative tip-sample position.

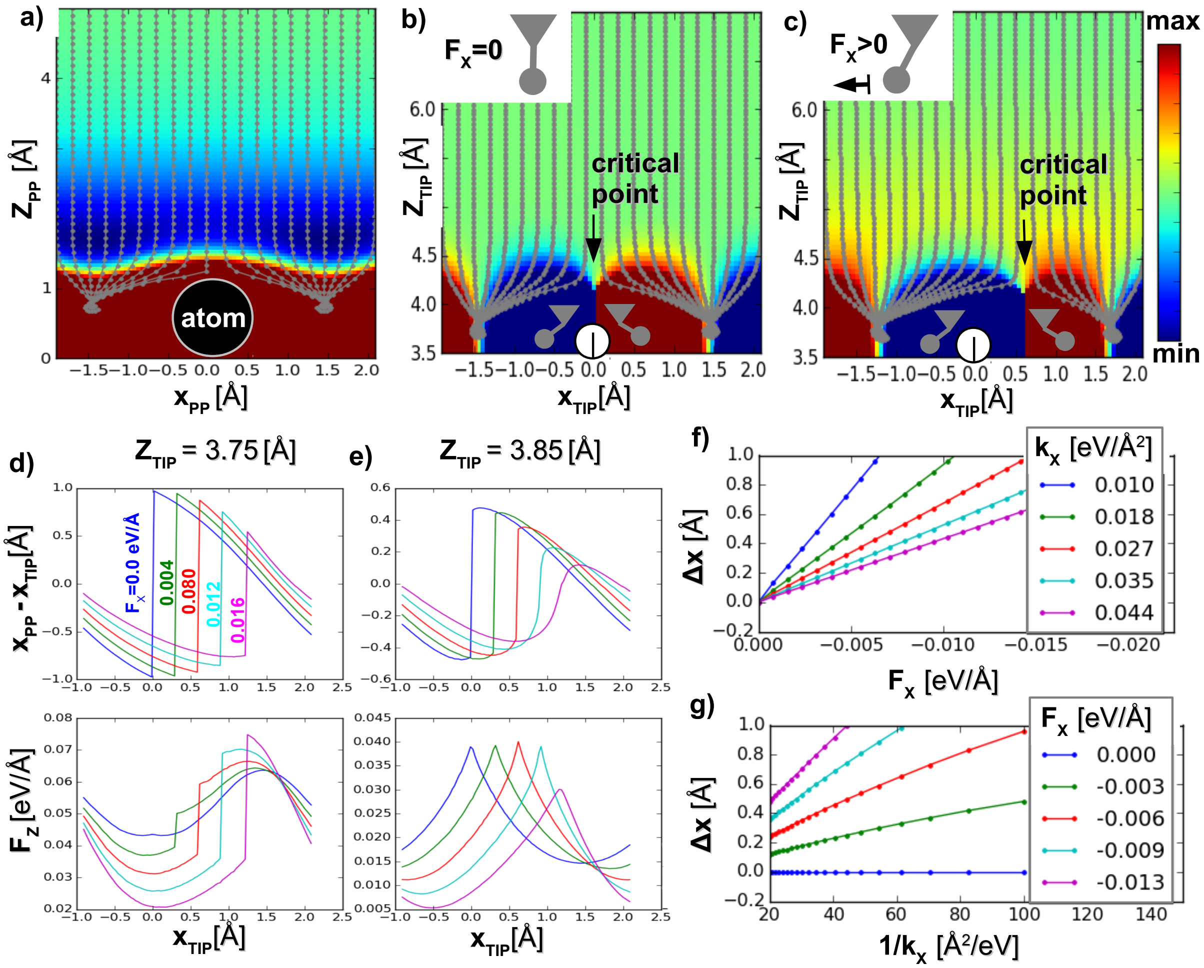

Supplementary Figure 2. **Linearity of image distortions.** a) Vertical (z) component of classical Lennard-Jones force field over the sample in the probe particle coordinates; gray lines depict trajectories of the probe particle movement upon tip approach. b) Deflection of probe particle d$x = x_{\mathrm{tip}} - x_{\mathrm{PP}}$ shown as color-code (red and blue tones represent bending right and left, respectively). The map is plotted in coordinates of the tip position x_{tip}. c) The same plot for the case when a constant lateral force F_x is added to the LJ force field; d,e) profile of the probe particle deflection dx (top) and corresponding measurable vertical force acting on tip F_z (lower) at very close tip-sample distance before (e) and after (d) the contrast inversion plotted for different values of the additive lateral force F_x. The important feature is the discontinuity observed in deflection dx, which moves uniformly with increasing lateral force F_x. f) The dependence of the apparent position of the discontinuity Δx on the lateral force F_x for various values of the bending stiffness k (top) and its dependence on the inverse bending stiffness $1/k$ for different values of the lateral force F_x. The plot reveals the linear dependence of the apparent shift Δx on the lateral force F_x according to the Hook's law.

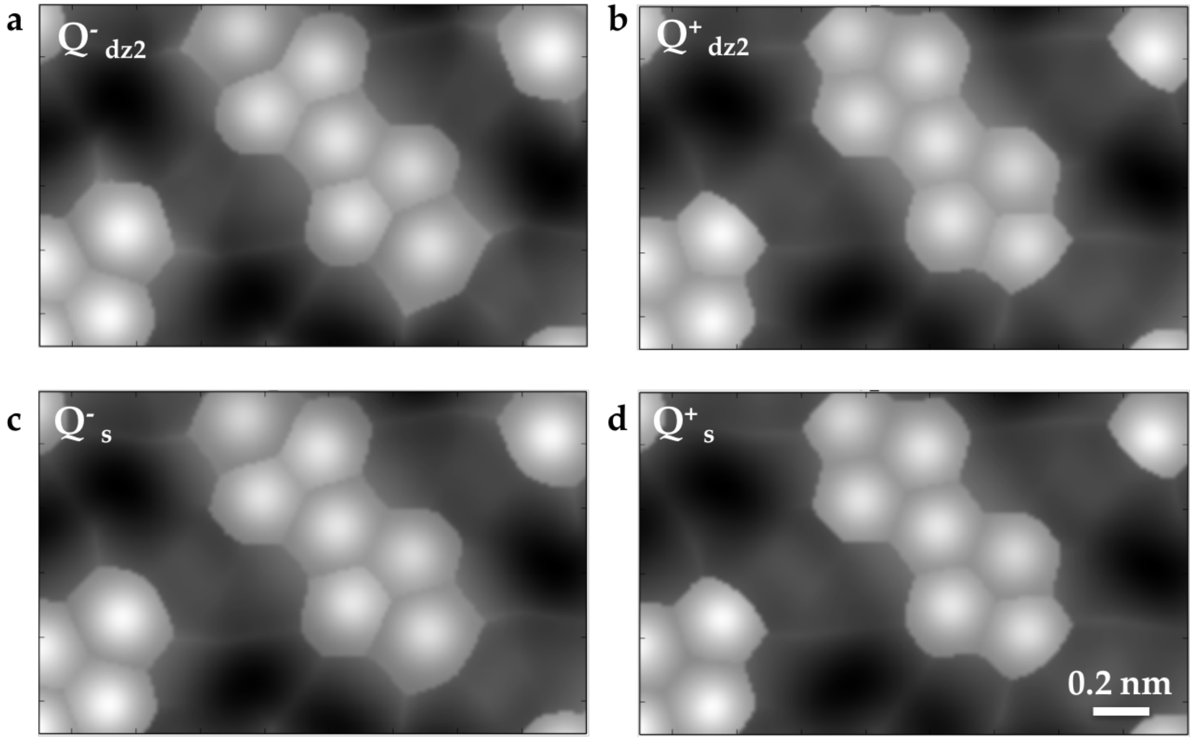

Supplementary Figure 3. **Effect of an effective charge distribution on the probe particle.** Comparison between simulated high-resolution AFM images of the PTCDA/Ag(111) at the same tip-sample distance using the same effective stiffness (k=0.20 Nm^{-1}) but different charge tip model (i.e. d_{z^2}-like quadrupole and s-like monopole model, respectively). Figures a) and b) displays the simulated AFM images with a d_{z^2}-like quadrupole model negatively and positively charged (Q_{dz2}=±0.3 eÅ^{-2}). Similarly, c) and d) displays the simulated AFM images using a s-like monopole model with negative and positive effective charged (Q_s=± 0.1 e).

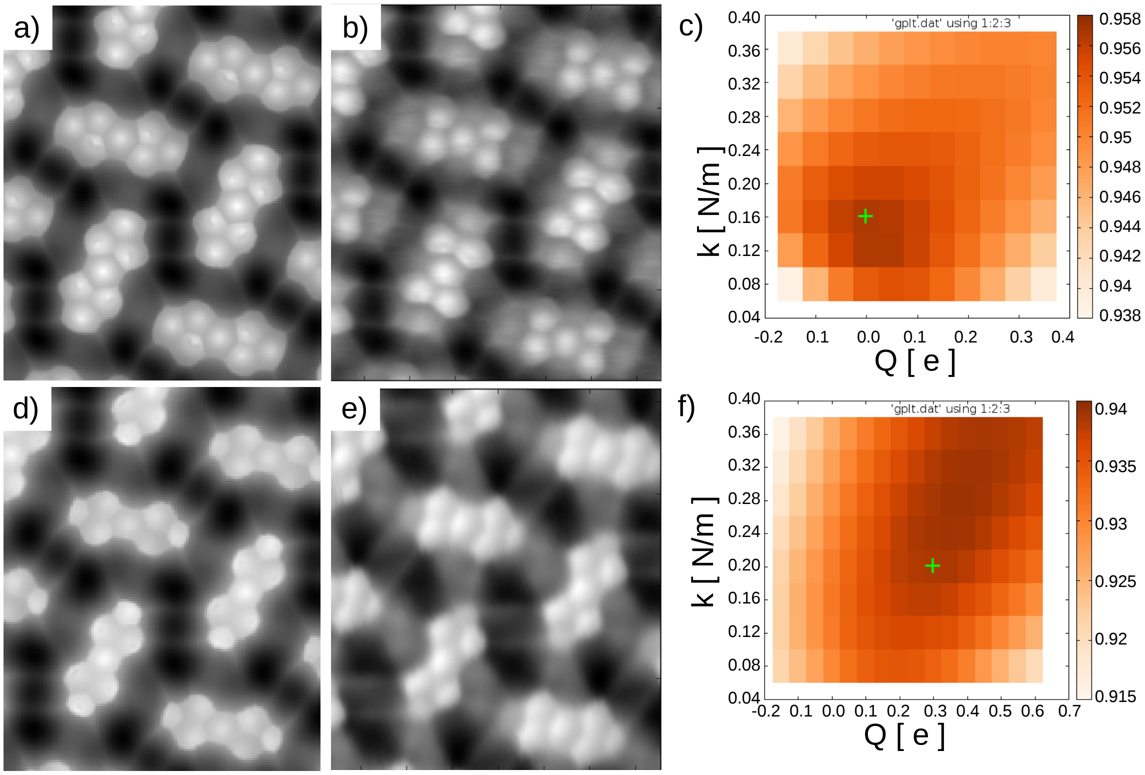

Supplementary Figure 4. **Estimation of effective K and Q for PTCDA** Two sets of frequency shift data are processed, registered and compared to the theoretically calculated maps. The input parameters (the probe particle charge Q and bending stiffness K) of the best-matching theoretical data sets represent an estimation of the probe particle characteristics in the experiment. a) theoretical image corresponding to tip A with k=0.16 Nm^{-1} and Q=0.0 e b) experimental image with tip A c) 2D map of linear correlation between theoretical and experimental image depending on stiffness and effective charge in mode d) theoretical image corresponding to tip tip B with k=0.20 Nm^{-1} and Q=+0.3 e e) experimental image with tip B f) 2D correlation map for tip B.

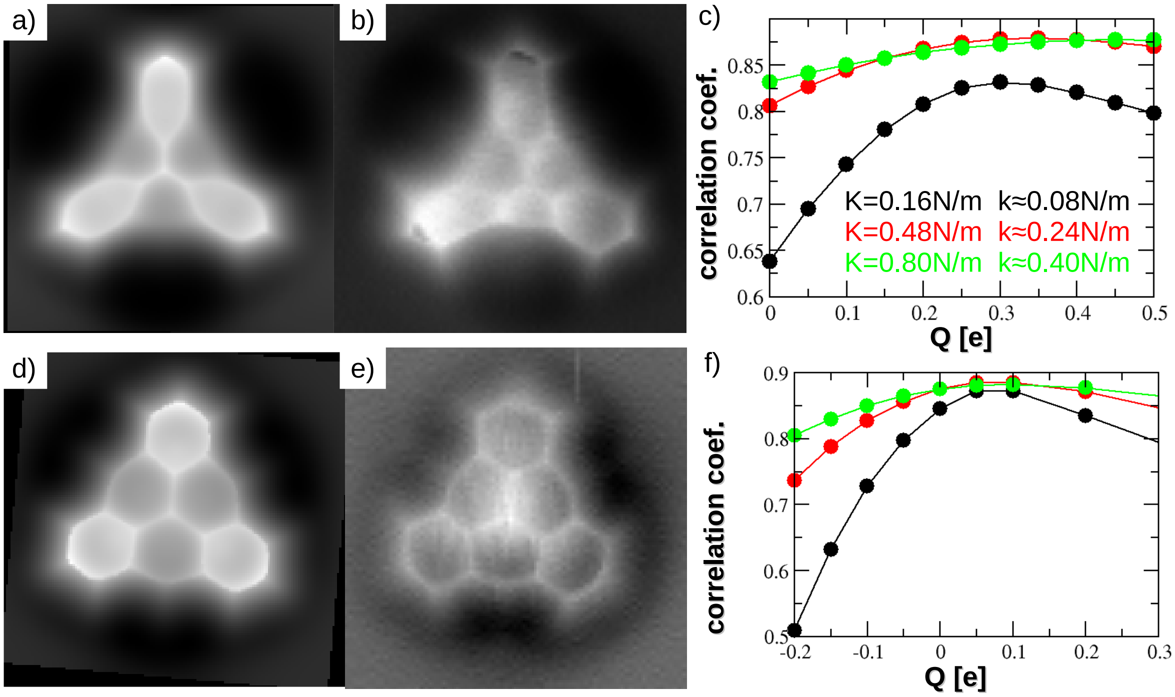

Supplementary Figure 5. **Estimation of effective K and Q for TOAT** a) Experimental Δf image of TOAT with Xe tip b) Simulated AFM image of TOAT with Xe tip and effective charge $+0.35\,\mathrm{e}$ c) linear correlation coefficient between experimental image (a) and simulated images with Xe tip different effective charge Q and stiffness k. d) experimental Δf image of TOAT with CO tip e) simulated AFM image of TOAT with CO tip and $0.0\,\mathrm{e}$ charge. f) linear correlation coefficient between experimental image (d) and simulated images with CO tip different effective charge Q and stiffness K.

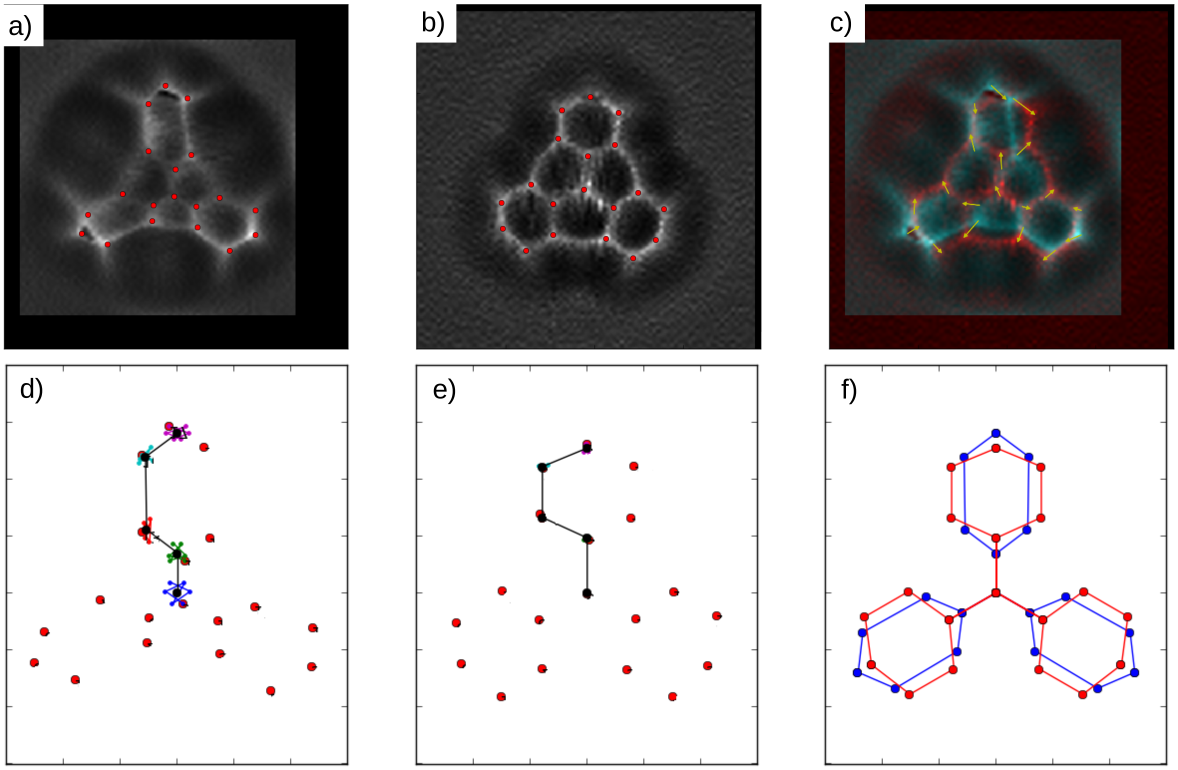

Supplementary Figure 6. **Extraction of image distortions for TOAT molecule** Manual assignment of feature positions and symmetrization of distortion vectors for TOAT molecule a) high-pass filtered Δf image of TOAT obtained with Xe tip and (b) CO tip. c) overlay of the two images (a) and (b) with corresponding distortion vectors between assigned features. The distortion is asymmetric due to tip asymmetry. d) symmetrization of features assigned to image (a) considering $C3v$ symmetry of TOAT molecule by projection into irreducible wedge and averaging. e) symmetrization of features assigned to image (b) by same means. f) apparent skeleton of TOAT molecule constructed from symmetrized features from (d) and (e).

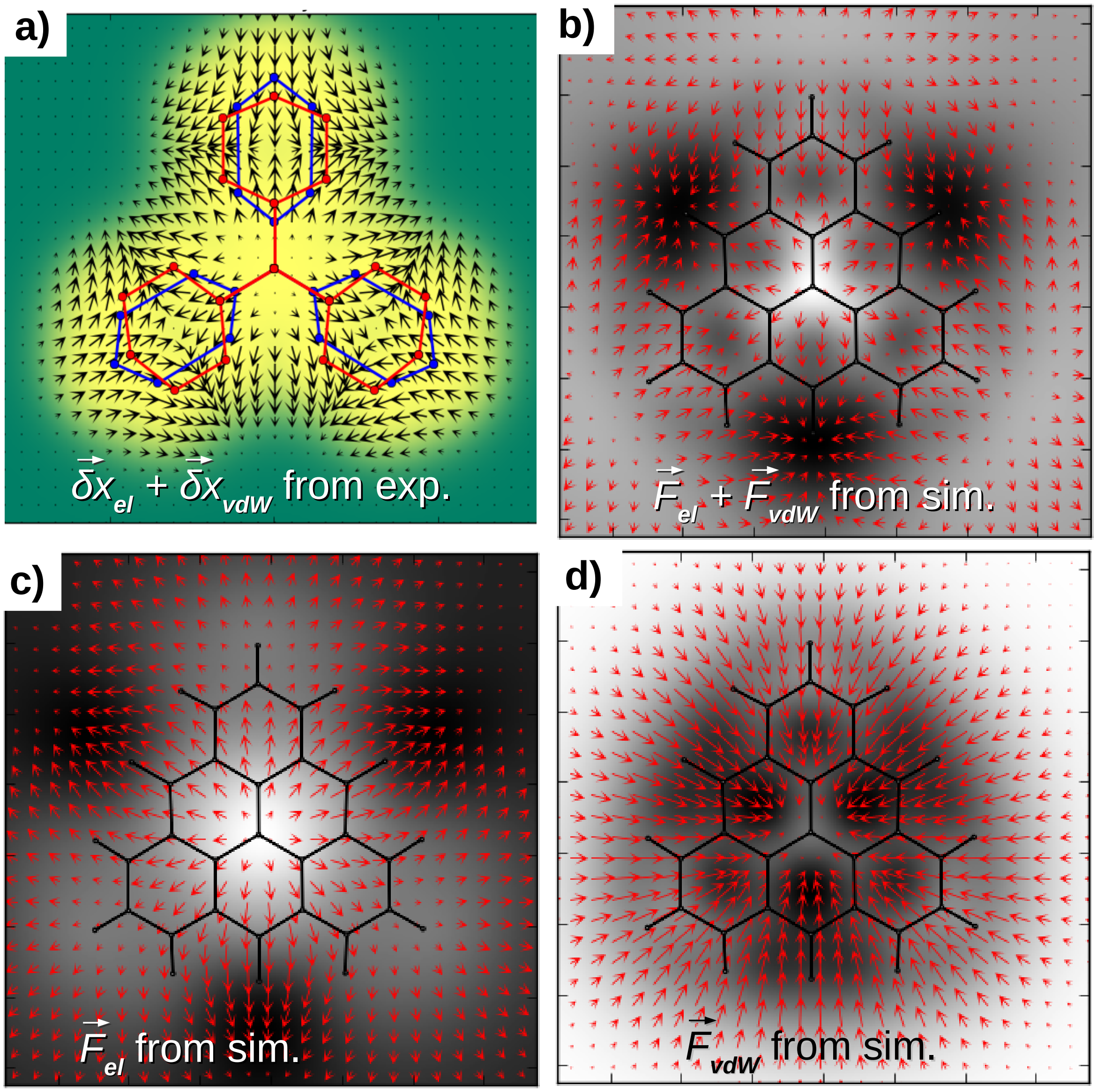

Supplementary Figure 7. **Subtraction of van der Waals component from distortion field on TOAT** Electrostatic and van der Waals component of the lateral force field and distortion for TOAT molecule: a) distortion field obtained from experimental images by interpolation of relative feature displacements, b) total force field used in numerical simulation, c) electrostatic component of the force field used in simulations obtained from DFT Hartree potential, d) Lennard-Jones component of the force field used in simulations, computed as a sum of pairwise interactions between atoms. Image b) is a linear combination of c) and d). The background depicts vertical component of the force field. The height z at which the fields were calculated corresponds to the onset of probe particle deflection.

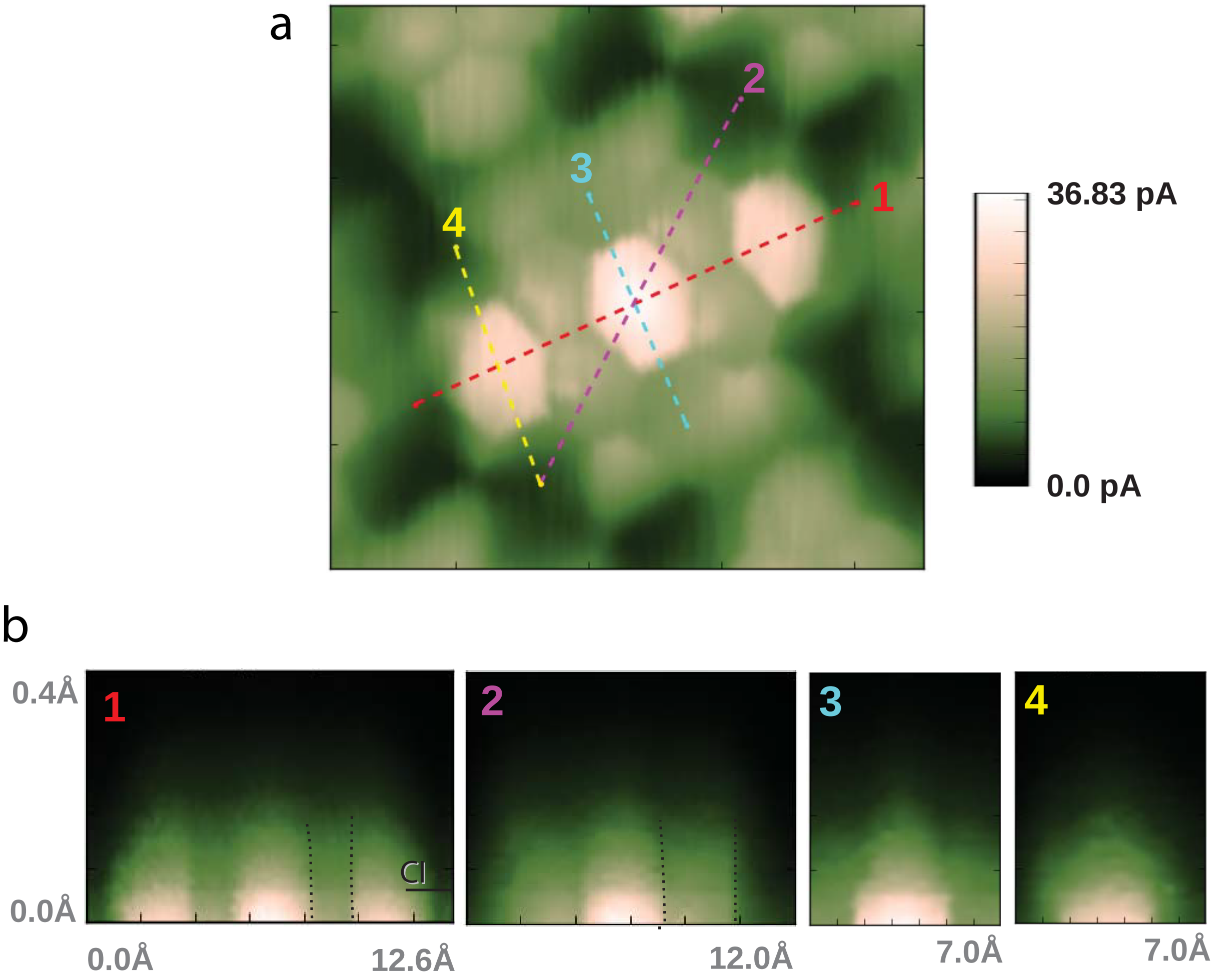

Supplementary Figure 8. **Sharp edges in STM** STM images and slices that indicate sharp edges caused by bending of the prope particle. **(a)** Current image obtained in constant height mode. **(b)** Slices at the lines indicaed in (a). The Figure shows the STM equivalent of some subfigures of Fig. 2 in the main text. It shows that also in STM images sharp edges are observed that could be used to determine the electrostatic field.

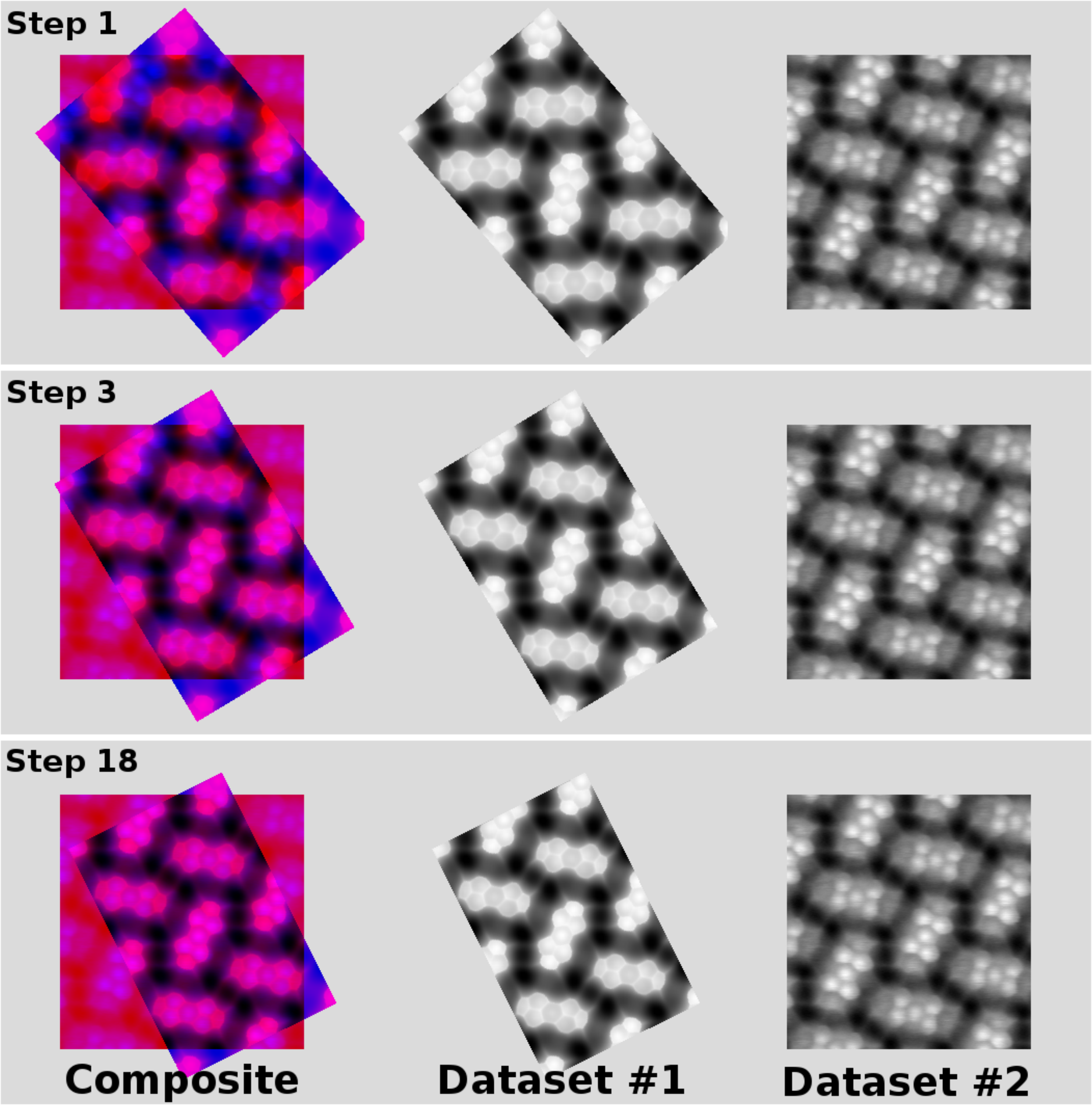

Supplementary Figure 9. **Data set registration procedure**: An example of the registration optimization. The two data sets are initially misaligned; by searching for the best linear Pearson correlation between the overlapping regions of the data sets, it is possible to find the optimum parameters of the transformation.

SUPPLEMENTARY NOTES

Supplementary Note 1. Hard Sphere Model: Where $\Delta\mathbf{x} \approx 2\mathrm{d}\mathbf{x}$ comes from, and why we may ignore $\mathbf{F}_{\mathrm{Pauli}}$

It was stated in the main text of the paper that the apparent shift $\Delta\mathbf{x}$ of an image feature is proportional to the lateral force, $\Delta\mathbf{x} = \gamma\mathbf{F}_{\mathrm{tot}}/K = \mathbf{F}_{\mathrm{tot}}/k$ for the in-palne components, where $k = K/\gamma$ is the effective lateral stiffness. The coefficient $\gamma \approx 2$ depends on van der Waals radii of the atoms. Below we will discuss where this relations comes from.

While the numerical simulations can easily find precise position of cusps which manifest itself as sharp edges on the experimental images for an arbitrary distribution of electrostatic field, Pauli repulsion and va der Waals, an analytic solution, although approximate, would provide better insight. Here we provide a simplified model, where Pauli repulsion of an atom is represented as an impenetrable hard sphere, and the lateral force (other than Pauli, e.g. electrostatic) is homogeneous. Due to the very steep onset of Pauli repulsion, this model reflects quite faithfully the behaviour of a more general force field used in numerical simulations, but unlike them it can be solved analytically using simple geometric considerations.

In the hard-sphere model, we completely neglect any attractive interaction (e.g. van der Waals). The repulsive interaction (Pauli repulsion) is represented by impenetrable hard spheres around atoms, which ensure that the probe particle cannot approach to a surface atom j closer than $R_{\mathrm{surf}} = R_{\mathrm{PP}} + R_j$ (where R_j is radius of the impenetrable sphere of the surface atom and R_{PP} of the probe particle). We assume that the probe particle is attached to the tip apex by a rigid radial bond of length R_{tip} (alternatively this constrain could be interpreted as Pauli repulsion between hard spheres of tip apex atom and the probe particle). Thus the probe particle can rotate around the tip base on a sphere of radius R_{tip}. A simplified 2D representation of the hard sphere model is depicted in Supplementary Figure 1. The probe particle is allowed to move on the blue or red circle. The probe particle is deflected by an angle $\alpha = \sin^{-1}(F_x/(K \times R_{\mathrm{tip}}))$ due to presence of the lateral force component F_x and the restoring lateral spring of the stiffness K.

A point of contact between the probe particle and the surface atom is defined on a contact of two spheres: blue/red of radius R_{tip} and gray of radius R_{surf} as depicted in Supplementary Figure 1. After the contact between the probe particle and surface atom, the probe particle begins to bend aside since the spheres are impenetrable. The critical point, corresponding to the sharp edge in AFM image, corresponds to the situation when angles $\alpha = \beta$, where angle β is defined in Supplementary Figure 1. In other words, this means that Pauli repulsion on probe particle from the tip and from the sample are in line, canceling each other.

Moreover, the component of the force originating from repulsion of the hard spheres that would be tangential to the spheres and that could therefore contribute to the probe particle deflection is zero by construction of the model. This observation justifies our assumption that the effect of $\mathbf{F}_{\mathrm{Pauli}}$ on image distortion can be neglected.

Therefore the equilibrium bending angle $\alpha = \beta$ is determined only from the balance between the restoring spring force and the force due to electrostatic field. This conclusion would hold for any spherically symmetric force field. Note that in the real situation, the repulsion is almost always strongly dominated by the closest atom. The cloud of electronic density around the atom is approximately spherical and therefore the Pauli repulsion force has also approximately spherical symmetry around the center of the closest atom. Only in hollow positions in between atoms, the Pauli repulsion deviates from this rule considerably (i.e. contributions from several atoms are comparable), however, this areas are not relevant for the formation of sharp edges.

The Pauli repulsion is the dominant force which defines the manifold on which the probe particle moves during its relaxation, but its particular value is not important for the determination of the bending angle or the apparent shift of features in images. Instead, the position of features in an image can be deduced from geometrical relations (triangle similarity) as depicted in Supplementary Figure 1. Here we can also see the origin of the proportionality constant γ between real bending stiffness K of the tip and effective stiffness K, as well as between the deflection of probe particle $\mathrm{d}x$ and the shift of an apparent position of observable features Δx:

$$\Delta x = \frac{R_{\mathrm{surf}} + R_{\mathrm{tip}}}{R_{\mathrm{tip}}}\mathrm{d}x \tag{1}$$

$$= \frac{R_{\mathrm{surf}} + R_{\mathrm{tip}}}{R_{\mathrm{tip}}}\frac{F_x}{K} \tag{2}$$

$$= \gamma\frac{F_x}{K} \tag{3}$$

$$= \frac{F_x}{k}. \tag{4}$$

After substitution into the relation parameters used in our numerical simulation ($R_{\rm tip} = 3.0$ Å and $R_{\rm surf} = 1.908 + 1.6612 = 3.5692$ Å) we obtain factor $\gamma = 2.19$. This value is very close to the factor γ =1.9 obtained from the fit of the results of our 2D simulation, considering that the hard sphere model is very crude approximation of Lennard-Jones force field. To conclude, we believe the factor $\gamma = 2.0$ gives a very good approximation to estimate the effective lateral stiffness k from the bending stiffness K.

Supplementary Note 2. The linear dependence of the image distortions $\Delta\mathbf{x}$ on the lateral force

In the derivation of equation (6) in the main text, we assumed that the apparent lateral distortions $\Delta\mathbf{x}$ of the sharp features present in high-resolution AFM images depend linearly on the lateral force. At the first glance, this assumption may seem to be natural. However both the Lennard-Jones force field as well as the lateral deflection d$\mathbf{x}$ of the probe particle with respect to tip apex depend non-linearly on tip-surface separation.

For this reason, it is necessary to discuss the detailed mechanism, which justifies the linear dependence of the apparent distortion $\Delta\mathbf{x}$ of the sharp features in AFM images on the lateral component of $\mathbf{F}_{\rm tot}$. In order to illustrate this mechanism, we use a simplified 2D model (in x, z coordinates only). Let us consider a scan with a functionalized tip over an infinite chain of atoms along a x-axis consisting of carbon atoms equidistantly separated by 2.9 Å. Note that this distance corresponds approximately to two carbon atoms on the opposite sides of a benzene ring.

Supplementary Figure 2b shows evolution of the deflection dx of the probe particle with respect to the tip apex along tip-approach towards surface for different lateral positions of tip $x_{\rm tip}$. The lateral deflection dx of the probe particle to the left and right is depicted in blue and red tones, respectively, reflecting positive and negative sign of d$x = x_{\rm tip} - x_{\rm PP}$. We see clearly that the deflection dx depends non-linearly on the tip-height $z_{\rm tip}$ (as can be seen from both the gray-scaled trajectories and from the color-code in Supplementary Figure 2b), but the position of the sharp discontinuous boundary between the left and right bending (blue and red region) does not depend on the tip-sample distance $z_{\rm tip}$. This is due to the symmetry of the chosen system (the infinite chain of equidistant carbon atoms), which does not introduce any asymmetric lateral van der Waals force in far tip-sample distances. This means that the probe particle deflection dx is zero ($F_x = 0 => \Delta x = 0$) in the far distance. Consequently the saddle point of the potential energy surface is positioned symmetrically right on top of the center of carbon atoms.

In Supplementary Figure 2c we repeated the same simulation but with an additional lateral force component F_x pointing along x-axis, which can represent e.g. the electrostatic interaction between a charged probe particle and local surface electrostatic potential. We assume that this additional lateral force F_x induces an extra lateral deflection dx' of the probe particle, which is linearly proportional to F_x according to Hook's law d$x' = F_x/K$, where K is the lateral bending stiffness of the probe particle attached to the tip base. Consequently the two coordinate systems of tip ($x_{\rm tip}$) and the probe particle ($x_{\rm PP}$) are now shifted with respect to each other and the characteristic sharp feature in AFM images appears at different positions when measured by a scanning probe.

Supplementary Figure 2d,e display a profile of the deflection dx as function of the tip position $x_{\rm tip}$ in two very close tip-sample separations $z_{\rm tip} = 3.75$ Å (d) and $z_{\rm tip} = 3.85$ Å (e) for different lateral forces. Lower images represent the vertical component of the force (F_z) to provide a direct connection to measured AFM images. We deliberately choose these two distances (e) because they represent situation before (e) and after (d) so called *contrast inversion* in AFM image [1].

As you can see from the dx profile after the contrast inversion, shown in Supplementary Figure 2d, the position Δx of the discontinuous jump from negative to positive value of the probe particle deflection (left to right) moves linearly with the acting lateral force F_x. The discontinuities in the dx profile also correspond to discontinuities in F_z rendering sharp features in high-resolution AFM images [1]. We can find similar scenario in Supplementary Figure 2e before the contrast inversion. Nevertheless, in this case, F_z exhibits only sharp ridges instead of the discontinuous jumps at the position of the dx-discontinuities. Before the contrast inversion, you can also see that the dx is not completely discontinuous at the position where the surface feature is imaged. Thus the precise position of the feature is difficult to be localized. For that reason we find our method best-suited for images measured in very close distances after passing the contrast inversion point.

Finally, Supplementary Figure 2f and Supplementary Figure 2g show the dependence of the relative position of the sharp features Δx (i.e. position of sharp edges in the high-resolution AFM images) with respect to the different lateral force components F_x and the inverse bending stiffness $1/K$. The result of these simulations clearly shows that the position of the discontinuous features Δx depends linearly on both F_x and $1/K$ for a broad range of values, and thus it can be described by the Hook's law.

Supplementary Note 3. Relation between image distortion and lateral electrostatic force field: a detailed derivation

In this section, we explain the relation between the image distortion of characteristic sharp contours in AFM/STM images and a lateral electrostatic force field F_{el} acting on an apex of functionalized tip.

From equation (5) in the main text we obtain for the surface electric field:

$$\mathbf{E}_{\mathrm{S}} = \frac{k}{Q_{\mathrm{A}} - Q_{\mathrm{B}}}\delta\mathbf{x} = \frac{k}{\delta Q}\delta\mathbf{x}. \tag{5}$$

From this we can thus obtain $\mathbf{E}_{\mathrm{S}}$ from the difference in charge between the tips (δQ) and the effective lateral stiffness (k). Note, however that for this case tips A and B only differ in effective charge. Other, more practical cases, will be eluded below.

1. *One tip has negligible effective charge and lateral van der Waals force is negligible*

If the high-resolution AFM/STM images were acquired with two different tips A and B, where both effective charge Q and effective stiffness k differ, the electrostatic potential can still be extracted under certain conditions. For example, if the effective charge on one of the tips is negligible (i.e. $Q_{\mathrm{B}} \approx 0$), we can rewrite equation (4) from the main text as:

$$\delta\mathbf{x} = \frac{Q_{\mathrm{A}}}{k_{\mathrm{A}}}\mathbf{E}_{\mathrm{S}} + \delta\mathbf{x}_{\mathrm{vdW}}, \tag{6}$$

where $\delta\mathbf{x}_{\mathrm{vdW}}$ means a residual component of the van der Waals lateral force field rescaled by the effective lateral stiffness k, defined as:

$$\delta\mathbf{x}_{\mathrm{vdW}} = \frac{\mathbf{F}_{\mathrm{vdW,A}}}{k_{\mathrm{A}}} - \frac{\mathbf{F}_{\mathrm{vdW,B}}}{k_{\mathrm{B}}}, \tag{7}$$

consequently the local electrostatic field $\mathbf{E}_{\mathrm{S}}$ can be obtained from the expression:

$$\mathbf{E}_{\mathrm{S}} = \frac{k_{\mathrm{A}}}{Q_{\mathrm{A}}}(\delta\mathbf{x} - \delta\mathbf{x}_{\mathrm{vdW}}); \tag{8}$$

What remains to be determined, is the estimation of the residual van der Waals component $\delta\mathbf{x}_{\mathrm{vdW}}$ present in equation (8). Here we have two options. First, for certain systems (such as flat densely packed molecular layers or large organic flat molecules such as graphene flakes) van der Waals potential is typically slowly varying on sub molecular scale and lateral van der Waals force component can be neglected; consequently the equation (8) reduces to:

$$\mathbf{E}_{\mathrm{S}} \approx \frac{k_{\mathrm{A}}}{Q_{\mathrm{A}}}\delta\mathbf{x}. \tag{9}$$

In the case of small single molecules, when lateral van der Waals force component cannot be neglected, the residual vector field $\delta\mathbf{x}_{\mathrm{vdW}}$ can be calculated on a dense grid using numerical modeling employing empirical potentials and subtracted from equation (8). We have to admit that in this case, a precise quantitative determination of the electrostatic potential might be problematic due to approximations adopted during evaluation of the $\delta\mathbf{x}_{\mathrm{vdW}}$ component.

This case corresponds to imaging TOAT molecule on Cu(111) surface with Xe and CO-functionalized tips discussed in the main manuscript. Indeed the comparison of the high-resolution AFM images acquired with CO-functionalized tip with numerical simulations for different effective charges reveals that the effective charge present on CO-tip can be neglected. The method still can provide valuable information about charge distribution on molecules with atomic resolution.

Tip A and B differs only by stiffness; lateral van der Waals force is negligible

Another limit case consist of lateral forces acting on the probe particle being the same for both tips ($\mathbf{F}_a \approx \mathbf{F}_b = \mathbf{F}$) while the lateral stiffness differs ($K_\mathrm{A} \neq K_\mathrm{B}$). This case may correspond to a tip decorated by a particle with a strong internal electric field (characterized by the effective charge Q). Typically such internal charge of the attached molecule is only weakly modified by details of attachment to the tip apex, while the effective lateral stiffness can depend strongly on the geometry of a tip apex (different chemical species present on the apex of a metallic tip).

In this limit, the lateral force $\mathbf{F}$ can be obtained from $\delta\mathbf{x}$ as follows:

$$\mathbf{F} = \frac{k_\mathrm{A} k_\mathrm{B}}{k_\mathrm{A} - k_\mathrm{B}} \delta\mathbf{x}. \tag{10}$$

If we further assume that lateral force $\mathbf{F}$ is dominated by the electrostatic component $\mathbf{F} \approx Q\mathbf{E}_\mathrm{S}$ (i.e. van der Waals is negligible) then we arrive to an expression for the local electrostatic field $\mathbf{E}_\mathrm{S}$:

$$\mathbf{E}_\mathrm{S} = \frac{k_\mathrm{A} k_\mathrm{B}}{(k_\mathrm{A} - k_\mathrm{B})Q} \delta\mathbf{x}. \tag{11}$$

As discussed above, the lateral van der Waals force can be ignored on large flat and densely packed molecular surfaces or graphene flakes, because in such system van der Walls potential is rather homogeneous along lateral directions.

Supplementary Note 4. Effect of an effective charge distribution on the probe particle

In the manuscript, we consider monopole distribution of the effective charge on the probe particle. We should admit that in some cases a dipole (p_z) or quadrupole (d_{z^2}) distribution can be more appropriate. For instance for a CO-functionalized tip, our recent charge-distribution analysis points to the d_{z^2}-like character. However, we have checked that tips using monopole and quadrupole charge distribution provide very similar sub-molecular contrast save some tiny features mostly observed in intermediate and far tip-sample distance regimes, as shown on Supplementary Figure 3. A detail analysis about the calculations using different charge models on the probe particle will be published elsewhere. Multipole expansion to some low order would be useful to characterize the charge distribution mostly in a far field, where the distance between interacting particles were much larger than the size of the particles, or in a homogeneous field. Contrary to this, the electrostatic field of the sample is very inhomogeneous. Namely, it decays quickly with the z-distance. For the electrostatic interaction in near field, the most important contribution originates from local partial charges on the surface of those atoms which make direct contact between tip and sample. This property is often used in chemistry to rationalize intermolecular interaction, by plotting electrostatic potential map on top of electronic density isosurface. In the case of the CO-functionalized tip, the partial charge at the protruding end of the CO molecule is particularly relevant for the resulting image appearance. Note, Ellner et al. [2] came very recent publication to the same conclusion. Moreover, the character of the electric field on a CO-functionalized tip is not only affected by the multipole moments of the CO molecule but also by a charge transfer between CO and metallic tip. This may depend significantly on particular atomic and chemical structure of the metallic tip. However, detailed analysis of this effects is subject of an ongoing investigation beyond the scope of this paper.

The impact of a dipole or quadrupole present on the probe particle might be more profound if it were oriented in the xy plane rather than along the z axis. However, we are not aware of any functionalized tip currently employed with a prominent lateral dipole/quadrupole charge distribution. Commonly used functionalized tips such as CO, Xe, Cl can be described either as monopole or dipole/quadrupole oriented along the z-axis.

One can also ask how the deflection of the probe particle affects the situation by deflecting an originally vertical dipole, thus giving it a lateral component. Even though the relaxations at very close distance (i.e. beyond contrast inversion) can be quite substantial (>1 Å), this is irrelevant for apparent position of edges on the images. Since the position of a bifurcation (cusp) from which the edge originates is established at the onset of repulsion (contrast inversion). Therefore the only lateral component of dipole/quadrupole that may be relevant will arise due to bending of the tip termination by electrostatic and van der Waals forces before the onset of Pauli repulsion. This effect can introduce minor nonlinearity into the relations. However, the lateral deflection of the probe particle is always well bellow 0.5 Å, while a lever distance between the probe particle and metallic base tip atom is at least 4.0 Å. Therefore the lateral component of the dipole oriented along the lever can be at most 12 % of the total dipole. This is smaller than other uncertainties involved in the analysis.

Finally, the proximity of the probe particle may also affect the charge distribution on the inspected molecule. The charge distribution in organic molecules is mostly determined by chemical bonding. The energy connected with this charge redistribution within the molecule is therefore on the order of the binding energy of covalent bonds. We do not expect that forces and energies involved in this kind of measurement with a flexible probes (F $<$ 1 nN, E $<$ 0.1 eV) could considerably affect electron redistribution within the molecule. Thus we do not consider that this effect plays dominant role in the imaging mechanism.

SUPPLEMENTARY METHODS

Correlating the experimental and theoretical data sets to obtain the probe characteristics K and Q

The mechanical probe particle model [1, 3] (avaible opensource at https://github.com/ProkopHapala/ProbeParticleModel ; see also webpage http://nanosurf.fzu.cz/ppr/) can generate frequency shift maps in three dimensions. The model uses as an input the model of the optimized structure and corresponding Hartree potential obtained from the total energy DFT calculations and two additional parameters - the effective charge of the probe particle (Q) and the stiffness of the particle on the tip (K). As the two parameters affect the contrast and proportions of the molecules, they can be fitted to match the experimental data. The fitting can be divided into four basic steps:

1. A theoretical three-dimensional frequency shift is generated for one set of parameters.
2. The offsets in $z-$distance of the experimental and theoretical data sets are determined according to the $z-$position of the frequency shift minimum for the centers of the molecules.
3. The calculated images are periodically replicated according to the lattice vectors and registered onto the experimental data set, according to the registration procedure described above. The transformation parameters are stored.
4. Finally, the transformation is performed on other theoretical frequency shift maps generated for a range of the input parameters K,Q. The correlation with the experimental data is recorded for each. Thus, a dependence of the correlation coefficient on the input parameters of the model can be derived and the parameter values, which achieve the best match, could be identified.

However, it should be noted that precision of linear correlation similarity criterion and the classical force field simulation model used for production of theoretical images is sufficient just for approximate determination of parameters K,Q. The exact value of K,Q is still uncertain within a rather broad and shallow valley of the best fit (see Supplementary Figure 4c,f) .

Estimation of the image distortion form high-resolution AFM images of TOAT molecule

In the case of single TOAT molecule, the situation is too complicated for a reliable application of automated procedures used in the case of the PTCDA/Ag(111) for finding distortion of images, especially due to the lack of sharp features in the intermolecular region. Here we opt for a manual approach, where features of the apparent position of atoms (as vertexes) were assigned manually. Supplementary Figure 6a,b represent high-pass filtered AFM images obtained with Xe (a) and CO tip (b), respectively. However, the resulting distortion displayed in Supplementary Figure 6c shows certain asymmetrical pattern not compatible with the inherent $C3v$ symmetry of TOAT molecule. This asymmetry is most likely caused by an asymmetrical configuration of CO/Xe on the tip base [1]. Thus we filtered out the asymmetry by imposing the $C3v$ symmetry manually. To do that, we fold all corresponding points to the same irreducible wedge (see Supplementary Figure 6d,e) and taking their average position. After this symmetrization we obtained two apparent molecular skeletons depicted in Supplementary Figure 6f. Next, we used radial basis function (RBF) technique, which is commonly used for interpolation of a non-uniformly distributed data. In our implementation the resulting deformation field was renormalized by sum of all contributing basis functions:

$$\Delta\mathbf{x}(\mathbf{x}) = \frac{\sum_i \Delta\mathbf{x}_i \phi(\mathbf{x} - \mathbf{X}_i)}{O_{\text{cut}} + \sum_i \phi(\mathbf{x} - \mathbf{X}_i),} \tag{12}$$

where $\mathbf{X}_i$ denotes the assigned position of the sharp features (data points) and $\Delta\mathbf{x}_i$ is the distortion for this particular feature. As the the radial basis function ϕ we used positive function $\phi(\mathbf{R}) = \exp(-2|\mathbf{R}|)/|\mathbf{R}|$ (where $\mathbf{R}$ means the

position in image space in Å). Threshold parameter $O_{\mathrm{cut}} = 0.01$ was introduced to cut-off unreliable values interpolated far from the data points. Note that this method provides credible values of the distortion for points very close to the data points and between them, but it fails outside of a molecule. Therefore we cannot access any information about the electrostatic potential outside of the molecule using this method.

Subtraction of van der Waals component from distortion field on TOAT

As can be seen comparing Supplementary Figure 7a and Supplementary Figure 7b, the deformation field obtained from differences between feature positions in the two HR-AFM images closely resembles the theoretically predicted total lateral force field used in our simulations. The effect of the van der Waals component is visible only on peripheral benzene rings, where the direction of distortion is reversed.

In order to extract just the electrostatic component, we subtracted the computed van der Waals component. This component can be easily computed by summing pairwise component of Lennard-Jones force field between atoms. However, to perform this subtraction we need to estimate a proportionality factor β in $\delta\mathbf{x}_{\mathrm{vdW}} = \beta\mathbf{F}_{\mathrm{vdW}}$. In general, the difference of distortions due to van der Waals can be computed as

$$\delta\mathbf{x}_{\mathrm{vdW}} = \left(\frac{C_{\mathrm{CO}}}{k_{\mathrm{CO}}} - \frac{C_{\mathrm{Xe}}}{k_{\mathrm{Xe}}}\right)\frac{\mathbf{F}_{\mathrm{vdW,Xe}}}{C_{\mathrm{Xe}}} = \beta_{\mathrm{Xe}}\mathbf{F}_{\mathrm{vdW,Xe}}, \tag{13}$$

where $k_{\mathrm{Xe}} = K_{\mathrm{Xe}}/\gamma$ and $k_{\mathrm{CO}} = K_{\mathrm{CO}}/\gamma$ denote effective stiffness of the respective tips, C_{Xe} and C_{CO} are tip-dependent pre-factors of van der Waals interaction strength, and $\mathbf{F}_{\mathrm{vdW,Xe}}$ ($\mathbf{F}_{\mathrm{vdW,CO}}$) is the van der Waals force computed for the Xe (CO) tip.

From our fits it seems that bending stiffness of both tips is rather similar $K = 0.48$ Nm^{-1}, therefore we use common effective stiffness $k = 0.24$ Nm^{-1}. Then the expression simplifies to:

$$\delta\mathbf{x}_{\mathrm{vdW}} = \frac{1 - C_{\mathrm{CO}}/C_{\mathrm{Xe}}}{k}\mathbf{F}_{\mathrm{vdW,Xe}}, \tag{14}$$

The ratio of van der Waals interaction strengths $C_{\mathrm{CO}}/C_{\mathrm{Xe}}$ can be estimated by comparing binding energy ε_{ij} of Lenard-Jones dimers according to relation $\varepsilon_{ij} = \sqrt{\varepsilon_{ii}\varepsilon_{jj}}$. In our simulations we use $\varepsilon_{Xe,Xe} = 0.024$ eV and $\varepsilon_{O,O} = 0.009$ eV, which produce $C_{\mathrm{CO}}/C_{\mathrm{Xe}} = \sqrt{0.009/0.024} = 0.61$. This leads to the value of $\beta_{\mathrm{Xe}} \approx 26$. To check the robustness of estimation of the parameter β, we also tried to vary its value from $\beta = 20$ up to $\beta = 80$. We found that in this regions the overall shape is similar. In particular, for this interval of β the reversed direction of distortions of peripheral rings is diminished.

Data set registration procedure

It is necessary to perform a set of transformations to bring the data to a common coordinate system. We implemented a registration procedure, which could handle both 2D and 3D data sets.

In general, the task of the data set *registration procedure* was to find a minimal linear geometrical transformation, which would translate (xyz coordinates), expand/contract (in xy-directions only), rotate and distort (linear) all of the data set slices at once, to achieve the best possible match with the other. All actions have to be performed simultaneously on all the channels of interest, in order to preserve the data set integrity. As a measure of the "goodness" of the match between any two registered sets, we used a simple linear Pearson correlation of their overlapping regions on the selected channel (current or frequency shift). The initial values of the transformation parameters were manually set to a nearly matching situation. Afterward an algorithm optimized the values to reach the maximum correlation. The optimization algorithm used a simple gradient method with a decreasing step size. In all the cases, the convergence and the global maximum of correlation was easily achieved (see Supplementary Figure 9).

The procedure works very well on periodic structures, since they naturally contain a lattice, which provides anchoring points for matching with another data of the equal structure. This avoids the need for a precise distance and angle calibration, because the lattice serves as a reference itself. However, on AFM/STM images of single molecules, some parameters have to be restricted, e.g. fix the zoom at one level and disable any distortion, to maintain the physical distances in the data as it had been measured.

Evaluation of the electrostatic potential from the distortion vector field

Simple integration of the distortion vector field does not provide satisfactory results, due to small errors in the distortion vectors. Therefore, an adequate approach is a *fitting* of the vector field by a potential. We used a least-square minimization method by iterative blind random variation (mutation) of the potential. The resolution of the reconstructed potential is given by the resolution of the deformation vector field, in our case $3\times$ smaller than the resolution of the original images.

The algorithm takes the original vector field as the input and iteratively determines the potential in the following steps:

1. A potential is set to an array of random values and a vector field is calculated from it by finite differences.

2. The sum of squares is evaluated for the difference between calculated and the input vector fields. This sum of squares is remembered as a reference value.

3. Potential ϕ is modified by addition a randomly generated variational $\delta\phi$ and the field recalculated.

4. If the sum of squared differences improves, then a new potential and sum of squares is remembered, otherwise the step 3. is repeated.

This algorithm is repeated until the potential is recovered. In our case, the boundary conditions are set to zero and therefore the algorithm tends to over-relax the potential near the edges. Thus it is convenient to interrupt the procedure when the potential features reach a sufficient smoothness.

SUPPLEMENTARY REFERENCES

[1] Hapala, P. *et al.* The mechanism of high-resolution STM/AFM imaging with functionalized tips. *Physical Review B* **90**, 085421 (2014).

[2] Ellner, M. *et al.* The electric field of CO tips and its relevance for atomic force microscopy. *Nano Letters* **16**, 1974–1980 (2016).

[3] Hapala, P., Temirov, R., Tautz, F. S. & Jelínek, P. Origin of High-Resolution IETS-STM Images of Organic Molecules with Functionalized Tips. *Physical Review Letters* **113**, 226101 (2014).
